# Using Nominal Group Technique to Gather Recommendations in the Decision‐Making for Amputation Due to Diabetes

**DOI:** 10.1002/jfa2.70095

**Published:** 2025-11-03

**Authors:** Emilee Kim Ming Ong, Carolyn Murray, Susan Hillier, Ryan Causby

**Affiliations:** ^1^ Allied Health and Human Performance Unit University of South Australia Adelaide South Australia Australia

**Keywords:** amputation, decision‐making, diabetes, nominal group technique, person‐centred care

## Abstract

**Introduction:**

A lower extremity amputation has traditionally been considered as a last resort treatment option for people with a diabetes‐related foot ulcer (DFU). However, some people will opt for an earlier amputation to overcome the daily lifestyle challenges from ongoing conservative wound management. Even so, making the decision for non‐emergency amputation is challenging due to the lack of clear recommendations or evidence‐based resources. Therefore, this study aimed to gather recommendations from people with lived experience of a DFU or amputation, family members, health practitioners, and experts to guide decision‐making for amputation due to diabetes.

**Methods:**

Nominal group technique was used to gather and vote on recommendations to support people making decisions for amputation. This technique allows all voices to participate and inform ideas. Two separate cohorts were recruited, one group was comprised people with lived experience (of DFU or amputation due to diabetes) and family members (*n* = 4 participants). The other group consisted of health practitioners and experts (*n* = 5 participants). During these workshops, research findings from a previous scoping review (94 papers) and interviews with people with lived experience and health experts (*n* = 26) were presented to participants to gather iterative feedback. Recommendations were constructed using the previous findings and the participants' experience and expertise, which were voted on and later analysed using summative content analysis.

**Results:**

A total of 13 recommendations were established by the people with lived experience and their family members, and 15 recommendations from health experts. Seven categories were established from these combined recommendations which described the priority considerations: ‘Consider timing, with early discussions to move forward’, ‘Address every element of quality of life’, ‘Understand individual goals and priorities to make personalised decisions’, ‘Collaborate with support networks’, ‘Provide information and options’, ‘Communicate with respect and transparency’, and ‘Offer functional person‐centred systems’.

**Conclusions:**

The recommendations highlight the need for early and transparent discussions that prioritise individual goals, quality of life needs and collaboration with support networks, to enable person‐centred and evidence‐based decisions. These recommendations provide a foundation for the development of guidelines to support timely and informed collaborative decisions in the future.

## 1 Introduction

Diabetes is the primary cause of approximately 70% of lower extremity amputation (LEA) [1] and approximately 85% of these amputations are preceded by a diabetes‐related foot ulcer (DFU) [2]. People living with a DFU often encounter daily lifestyle interruptions including a strain on time and costs associated with wound management needs [3]. These can contribute to negative quality of life, prompting some people to request an LEA to move forward with the rest of their lives [4]. An LEA may be conducted in emergency situations when a DFU has become infected [5]. A decision for LEA in a non‐emergency situation for a DFU is not as straightforward, as the potential benefits from undergoing LEA must be considered for each person before making this life‐changing decision [6]. However, there is currently a lack of evidence‐based guidelines to support people in this situation [7]. A decision aid tool is available to support people undergoing foot amputation due to peripheral arterial disease [7] and clinical guidelines exist for the management of diabetes‐related foot infections and wounds [8, 9, 10]. The absence of evidence‐based guides impacts the ability for practitioners to provide consistent person‐centred recommendations for amputation, leaving people confused and anxious [6].

The nominal group technique (NGT) is a collaborative method for guideline development that is growing in use. For example, the World Health Organisation used NGT to integrate stakeholders' perspectives into healthcare recommendations for clinical guidelines for dementia and intensive care services [11]. The NGT has also been used to understand the needs of people with stroke and clinicians with power‐assisted exercise equipment [12]. This demonstrates the NGT as a robust method to explore amputation decision‐making and prioritise key considerations when making these decisions for LEA to establish future guidelines.

The NGT is a systematic and controlled process of group discussion to gather perspectives from groups, to prioritise and rank recommendations related to a topic [11]. The NGT aims to minimise pressures for conformity amongst participants common in group discussions, by establishing an environment where everyone is valued equally, and providing sufficient time to contribute their perspectives which is not achieved through Delphi surveys [13, 14]. Therefore, the NGT was an ideal method to use as it allows for group discussions and direct interactions amongst participants, which is not achieved through a Delphi process where participants remain anonymous and isolated in their responses [13]. The aim of this study was to gather and prioritise recommendations from people with lived experience (PwLE) of DFU or amputation, family members, health practitioners, and health experts about the decision‐making for amputation due to diabetes. The process was driven by outcomes from previous research including a scoping review of published evidence [15] and primary research of stakeholder experiences [6] for the decision‐making in diabetes‐related amputation.

**Research question:** Based on participants' experiences and opinions, the literature and primary data, what are the key recommendations from PwLE, family members, health practitioners, and health experts about the decision‐making for amputation due to diabetes?

## 2 Method

### 2.1 Ethical Considerations

Ethical approval was obtained from the Southern Adelaide Local Health Network (SALHN) (SALHN ethics LNR/23/SAC/85) and the University of South Australia Human Research Ethics Committees (reference number 205750). Written informed consent was obtained from all participants prior to the workshops and confidentiality and anonymity of participants was achieved by removing all identifiable information from data and in the reporting of the results.

### 2.2 Study Design

This study followed a modified NGT approach with mixed methods analysis of vote counting and content analysis of recommendations [16]. The purpose of the NGT workshops was to provide people with experience in making amputation decisions an opportunity to provide feedback and recommendations on previous primary and secondary research in decision‐making for amputation [6, 15].

### 2.3 Context and Background

This study builds on a body of work about the perspectives and priorities for decision‐making in PwLE, their family members, and health experts. Firstly, a scoping review about the clinical reasoning considerations and decision‐making processes described in the literature for non‐emergency amputation was completed and included in 94 papers [15]. Secondly, the gaps in research identified from this scoping review informed interviews conducted with people with a DFU or amputation, health practitioners, and research experts, to understand their decision‐making considerations [6]. The data from the 26 interviews were used to establish four themes with corresponding subthemes, which described key decision‐making considerations for amputation. The findings from both studies were presented during the workshop to inform the NGT process and recommendation generation.

### 2.4 Sampling and Recruitment

Because of the potential for a power imbalance between health experts, people with lived experience, and family members, two separate cohorts for the NGT workshops were recruited. The eligibility criteria for participants are included in Table 1.

**TABLE 1:** Eligibility criteria for participants.

	Population		Inclusion criteria	Exclusion criteria
Cohort one	PwLE	People living with diabetes‐related foot complications	– Currently receiving treatment from a high‐risk foot service for a DFU – History of LEA due to a DFU within the past 3 years. This includes people who have undergone a minor or major amputation	– Current or history of a foot ulcer or LEA not related to diabetes – Cognitive or communication disorder which would impact ability to participate in group discussions – Limited English proficiency
	Family members	Family members of PwLE	– Family member of a person currently receiving treatment from a high‐risk foot service for a DFU or with a history of LEA due to a DFU	– Limited English proficiency
Cohort two	Health experts	Health practitioners	– Currently providing DFU treatment in a tertiary care health service – Minimum 1 year of experience managing people who have had a diabetes‐related amputation	– Not currently providing DFU treatment – Limited English proficiency
		Experts	– People who are involved in public health departments or non‐government organisations promoting foot health, diabetes care or management of diabetes‐related foot complications	– No experience in managing or communicating with people who currently have a DFU or history of diabetes‐related LEA – Limited English proficiency

Abbreviations: DFU, diabetes‐related foot ulcer; LEA, lower extremity amputation; PwLE, people with lived experience.

Cohort one included PwLE (people living with a DFU or amputation), along with family members. The health service identified potential participants, and the principal researcher then approached them to provide information about participating in the study.

Cohort two were health experts which included health practitioners and other experts. Health expert participants from the previous study [6] plus new recruits, were purposively sampled through email correspondence and the research teams' professional networks.

A maximum of five participants for each cohort was sought to ensure everyone had adequate time to feedback to the group and ensure diverse perspectives [17]. A gift card honorarium was provided to participants.

### 2.5 Data Collection

The NGT workshops ran for one and a half hours and were audio recorded. Due to participant availability, the NGT workshops for cohort one were conducted on two separate occasions with different participants at a public hospital in metropolitan South Australia. The NGT workshop conducted with cohort two was conducted online via video call (Zoom Communications Inc., San Jose, California, United States) to enable participants from different countries, and a range of experiences to be captured.

The traditional NGT format [18] was modified to five phases to include a preliminary phase providing background information about the findings from the previous scoping review [15] and primary research [6]. This step was necessary as the NGT was building on the prior research. The principal researcher (EO) who was familiar with the amputation literature and had previous experience as a co‐facilitator in a NGT workshop was the primary facilitator. At least one other member of the research team joined the NGT workshops (RC, CM, and SH) acting as a co‐facilitator, taking notes, supporting discussions, and documenting the recommendations. The questions used to guide the workshops were collectively developed and refined by the research team and the principal researcher piloted the workshop structure with the research team in person and using the online platform prior to data collection to gain feedback on the structure and presentation technique.

#### 2.5.1 Phase 1 Introduction

**FIGURE 1:**
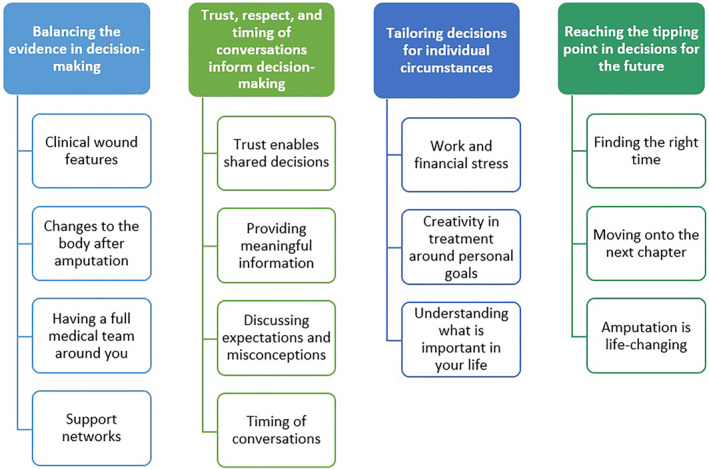
Themes and subthemes presented to cohort one.

**FIGURE 2:**
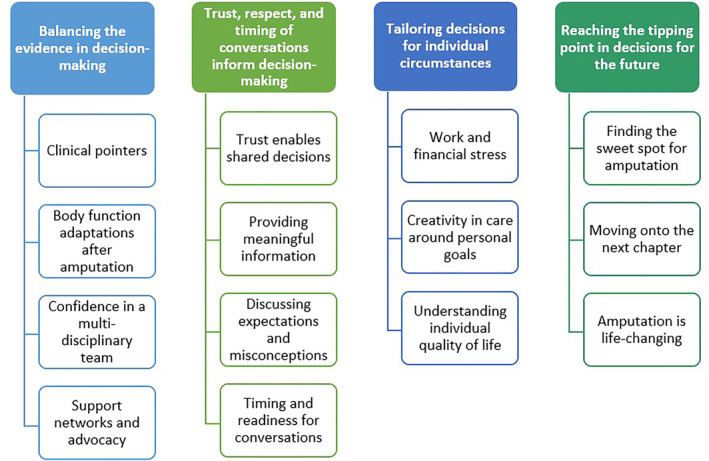
Themes and subthemes presented to cohort two [6].

The facilitator started by introducing the aim of the NGT and presenting the background information [6, 15] from the previous research. This information was adapted for cohort one to ensure suitability for people with varied health literacy levels (see Figures 1 and 2). An icebreaker activity was conducted to encourage sharing of ideas with the group, whilst familiarising the participants with the previous research. During this activity, participants were asked to review the list of themes (Figures 1 and 2) and select up to two themes or subthemes that resonated most with them. Participants were asked to share the reasons behind their decisions.

#### 2.5.2 Phase 2 Silent Generation of Ideas

Next, the facilitator presented the questions:

- To cohort one: ‘Looking at the themes, what are your key recommendations to support people like yourself who are making decisions for amputation?’
- To cohort two: ‘With consideration of the themes presented, what are your key recommendations for practitioners to consider to inform decisions for non‐emergency lower extremity amputation in people with a diabetes‐related foot ulcer?’

Participants had 10 min to individually create up to three recommendations. For cohort one, participants were asked to write each recommendation onto a piece of paper which was stuck onto a wall. For cohort two, participants shared their recommendations, which were displayed on a virtual whiteboard.

#### 2.5.3 Phase 3 Sharing of Ideas

Each participant had the opportunity to describe their recommendations with the group, sharing their ideas and explaining the rationale behind their recommendations.

#### 2.5.4 Phase 4 Group Discussion

Recommendations presenting a similar idea were grouped together on the wall (virtual or physical) and then discussed to ensure the statements accurately represented their intent.

#### 2.5.5 Phase 5 Ranking

Each participant was provided with three votes to distribute amongst the recommendations. These votes could be weighted to reflect their thoughts about a recommendation, for example, if they considered one recommendation extremely important then they could vote for that more than once. Physical voting was conducted using coloured stickers and the virtual voting was conducted in the online platform. Finally, participants were asked to identify any additional recommendations not from the prior work which they felt were missing to finalise the list. Group discussions about the list of recommendations were encouraged during this time to ensure the final ranked list reflected the decision‐making priorities of the group.

### 2.6 Data Analysis

The recommendations were analysed using summative content analysis to identify key priorities and enable comparison of data between the three NGT workshops [18]. Through this analysis, there was further interpretation of the recommendations, to identify similarities and differences across the dataset [19]. Two researchers (EO and CM) individually completed the following steps: 1. familiarised themselves with the data by re‐reading the list of recommendations and taking notes when listening to the audio recording from each workshop; 2. coded the recommendations through an inductive process to identify key areas from the two cohorts; 3. reviewed their list of codes from both cohorts and grouped together codes presenting a similar idea as a ‘category’. The research team then met to compare the codes and combine categories to finalise and rank the categories into key recommendations. A decision‐making wheel was constructed from the final list of seven categories. A summary of findings along with the decision‐making wheel were returned to the participants to confirm that the categories were reflective of their ideas.

#### 2.6.1 Rigour

Prior to the first NGT workshop, the research team shared their personal biases and experiences with each other to enhance reflexivity [20]. Credibility was achieved using two coders during data analysis and peer debriefing. Purposive sampling, reporting demographic details of the participants and rich descriptions of findings supports transferability to other contexts [21]. Confirmability was achieved through a reflexive journal used to document field notes after each workshop [21]. Providing thick descriptions of the NGT process ensured dependability in this research [20].

#### 2.6.2 Author Positioning

The principal researcher is a PhD candidate and podiatrist. The co‐authors are clinicians and researchers in podiatry, physiotherapy, and occupational therapy. The podiatrists have had experience in amputation decisions with people living with diabetes.

## 3 Results

There were two NGT workshops with cohort one and one workshop with cohort two. There were four participants in cohort one—two people with a DFU, one with a history of LEA, and one family member. Three of these participants were male, and one was female, with the mean age of people with a DFU or LEA 70.3 (SD 19; range 51–89) years.

In cohort two, there were three health practitioners and two experts, with four participants from Australia (located in the same state) and one located in the United States, ranging from four to 30 years of experience in their respective occupations. The demographic data of the participants are reported in Table 2.

**TABLE 2:** Demographic data of participants.

	Participant code	Age	Gender	DFU location	Duration of current DFU	History of amputation
Cohort one: PwLE and family members	C01	51	M	Lateral midfoot	2.5 years	No
	C02	71	F	Fifth digit	10 months	Yes (digit)
	C03	89	M	Hallux	6 weeks	No

	**Participant code**	**Age**	**Gender**	**Role**	**Employment status**	
	C04	25	M	Family member	Student	

	Participant code	Age	Gender	Country	Health practitioner (HP)/expert (E)	Occupation	Years of experience in occupation
Cohort two: Health experts	S01	38	M	Australia	E	Medical scientist	4
	S02	32	F	Australia	HP	Podiatrist	10
	S03	29	F	Australia	HP	Orthotist and prosthetist	7
	S04	55	F	USA	E	Professor of physical therapy and orthopaedic surgery	30
	S05	60	F	Australia	HP	Amputee nurse	15

Abbreviations: Expert, expert; HP, health practitioner; PwLE, people with lived experience; S, stakeholder; SA, South Australia; USA, United States of America.

### 3.1 Part 1: Group Discussion of Priority Themes in Amputation Decision‐Making (Phase 1)

There were six subthemes/themes identified by cohort one and seven subthemes/themes identified by cohort two (see Table 3).

**TABLE 3:** Priority themes and subthemes for participants (Phase 1).

Sub/themes	Selections
Cohort one: PwLE and family members	
Moving onto the next chapter	2
Changes to the body after amputation	1
Providing meaningful information	1
Trust, respect, and timing	1
Tailoring decisions to individual circumstances	1
Work and financial stress	1
Cohort two: Health experts	
Understanding individuals' quality of life	3
Finding the sweet spot for amputation	2
Support networks and advocacy	1
Amputation is life changing	1
Trust enables shared decisions	1
Work and financial stress	1
Timing and readiness for conversations	1

#### 3.1.1 Priority Considerations for Cohort One

In the two NGT workshops for cohort one, two participants selected ‘moving onto the next chapter’ as a key consideration in their decision‐making for amputation. One participant prioritised learning how to walk again after amputation (C01), whereas for another (C03), it was important to be able to ‘get on with life’ by undergoing an amputation.

‘Changes to the body after amputation’ was selected by one participant (C01) who was not concerned about the prospect of amputation, as he had a friend who had undergone multiple foot amputations and was able to manage well using a prothesis.

If it’s (amputation) got to happen, I wouldn’t worry about it too much as the prosthetics they’ve got out there are pretty good

(C01)

‘Providing meaningful information’ was selected by C02 as she had past experience of not being clearly informed that an amputation was going to take place. This participant described feeling dismissed by staff as ‘reading too much into it’ when requesting more information leaving her feeling ‘left in the dark’. C04 chose the subtheme ‘trust, respect and timing’ as they had trust in the multi‐disciplinary team to work collaboratively with them in the best interest for their grandfather. ‘Tailoring decisions to individual circumstances’ was chosen by C04 as she prioritised ensuring that the goals of care for a person with a DFU are understood by the team supporting decisions for amputation.

#### 3.1.2 Priority Considerations for Cohort Two

Three participants identified ‘understand individuals' quality of life’ as important because they recognised the ‘need to know’ a person's goals of treatment when making decisions—some people will try to maintain their limb for as long as possible, whereas others would prefer to ‘cut it off’ and move on. This theme was identified as the ‘cornerstone of our decision‐making’ (S05) because it supports practitioners to identify the tipping point in decisions. ‘Finding the sweet spot for amputation’ was selected by S02 and S03 as they recognised that for some people, intervening sooner with amputation benefitted positive outcomes post‐amputation.

### 3.2 Part 2: Recommendations Ranked by Votes (Phase 5)

#### 3.2.1 Cohort One Recommendations

Thirteen recommendations were gathered from cohort one. The two recommendations with the most votes [4] described identifying individualised goals for the person with a DFU. Three other recommendations, which centred around providing clear meaningful information and the presence of support networks each received two votes. Recommendations about options for moving beyond amputation and continuity of staff were ranked third, with one vote each (Table 4).

**TABLE 4:** Cohort one recommendations ranked by votes.

PwLE and family member recommendations	Votes
Ask about specific goals of the patient with regards to their care, ensuring that finances do not become a barrier to accessing good healthcare	4
Health practitioners ask me what is important in my life, when making the decision	4
Straight up information, explain but not in doctor's terms	2
Medical staff need to give correct information in language understood by the patient to answer questions asked, that is, LISTEN to the patient	2
When someone needs to make a decision about amputation, another family member or someone else also be present	2
Knowing options for moving onto the next steps after amputation	1
Continuity of staff and for medical staff to be aware of the patient's history	1
Medical staff need to learn how to treat patients as intelligent beings	0
Knowing which path you will go down	0
Ensuring that all decisions about a person's care are shared between the patient, the multi‐disciplinary team, and family members/friends to ensure the best outcomes	0
Health practitioners need to discuss/provide advice with how to move on with life (learn from previous experience)	0
Health practitioners hide nothing and provide complete honesty regarding the decision	0
Tailored information provided i.e., for different languages, different levels of literacy, so that a shared understanding can be met	0

#### 3.2.2 Cohort Two Recommendations

Fifteen recommendations were established by cohort two. Two recommendations that ranked equally highest (three votes each) described early discussions for amputation and quality of life considerations. Two recommendations received two votes each, one describing quality of life considerations before and after amputation, whilst the other described support networks. Five recommendations were ranked third with one vote each (see Table 5).

**TABLE 5:** Cohort two recommendations ranked by votes.

Health expert recommendations	Votes
Early discussions around amputation to include pre‐op counselling, rehabilitation specialists, and psychological supports	3
Consider the patient's quality of life now, and after amputation. This is not a one‐size‐fits‐all intervention, where many factors including pain, repeated hospitalisations, need to work, and support from family will determine how the person feels about losing a functional part of their body	3
Amputation should be considered when the individual's quality of life before the amputation is worse than the quality of life expected after amputation. Quality of life includes functional ability, ability to care for self and others, and overall health	2
Include the patient's networks. For many patients, decisions regarding amputation will need to consider how it will affect their friends, family, and others. People should be invited to consider and involve their network, reflecting how it will impact the community and how they will help care for the person long‐term. This is especially important for aboriginal and torres strait Islander people, and in addition to community they should also have an aboriginal health practitioner or other cultural wellbeing staff included to help with support	2
The prospect/possibility of non‐emergency lower extremity amputation in people with diabetes‐related foot ulcers should happen in a timely fashion in order to ensure patients have the opportunity to find the sweet spot for amputation	1
Take the time. From a clinician's perspective, these are some of the most complex disease patterns to manage. From a patient's perspective, this is a life‐threatening, life‐altering decision process. This discussion cannot be rushed, and in a non‐emergency situation, it should not be. For aboriginal and torres strait Islander people especially, these discussions need to allow for all questions and cultural considerations to be made, with ample time to absorb the magnitude and significance	1
Amputation timing should consider the sweet spot ‐ a point when limb salvage options have been reasonably exhausted, and the individual's health is not so compromised that recovery expectations are diminished. The clinician should be assisting the patient in accepting/readying themselves for amputation as a possibility	1
When considering/discussing amputation with a person (family, carer) with a diabetes related foot ulcer, consideration and care should be given to understanding an individual's current quality of life and what quality means to them. Explanations of the impact of amputation should be tailored to the above	1
It is ultimately the patient's decision	1
The patient's current mobility level and functional capacity to complete their ADLs are considered and documented	0
Individualised goal setting should be completed to establish the options and decisions that would improve their quality of life	0
Quality of life needs to be considered when amputation is to be considered as an option for patients. Patients need to be considered holistically—therefore need to consider functionally, emotionally, financially etc. This also links back to others in their life	0
Timing of conversation and finding sweet spot may improve outcomes for patient. For example, returning to home versus residential care facility, or prosthetic mobility versus wheelchair mobility	0
Conversations should begin early so that when the sweet spot/tipping point is reached the process can move forward in an efficient way that is maximising the patient's recovery potential	0
Cultural specificity should be considered and relevant	0

### 3.3 Part 3: Summative Content Analysis of Recommendations

The 28 recommendations provided in Tables 4 and 5 were analysed and aggregated using summative content analysis to reduce the data to seven categories (Table 6). These categories will be explained in order of most to least important.

**TABLE 6:** List of categories and frequencies.

Category	Frequency
Consider timing, with early discussions to move forward	13
Address every element of quality of life	9
Understand individual goals and priorities to make personalised decisions	8
Collaborate with support networks	6
Provide information and options	6
Communicate with respect and transparency	3
Offer functional person‐centred systems	2

#### 3.3.1 Consider Timing, With Early Discussions to Move Forward

Engaging in early conversations about amputation with people with a DFU and their support networks provided time to ‘understand the significance’ (S01) of the decision without rushing. Timing an amputation around the ‘sweet spot’ was recommended—a point where an amputation would enable improved outcomes and a person to ‘move forward to maximise their recovery’ (S04). Health practitioners were expected to take the lead in these conversations to assist with supporting readiness for amputation.

#### 3.3.2 Address Every Element of Quality of Life

It was recommended that early‐shared discussions be tailored around a person's quality of life expectations. The PwLE valued practitioners knowing what is important in their life, including financial implications and their goals. For health experts, pain, function, and ability to work influenced their understanding of quality of life. It was recommended that potential quality of life before and after an amputation be compared to assist decision‐making.

#### 3.3.3 Understand Individual Goals and Priorities to Make Personalised Decisions

This category described the need to position amputation decisions around individualised goals and priorities to facilitate personalised decisions. There is ‘no one‐size‐fits‐all intervention’ (S01) and therefore, decision‐making for amputation should incorporate ‘what is important in the person's life’ (C02). Ultimately, it was recommended to ‘consider the person holistically’ (S05), including financial barriers and cultural considerations.

#### 3.3.4 Collaborate With Support Networks

It was recommended to collaborate with support networks including family, friends, multi‐disciplinary teams, and cultural community groups in the decision‐making process; ensuring they are present and involved in decisions for amputation and recognising that a decision for amputation has impacts on these support networks as well.

#### 3.3.5 Provide Information and Options

People wanted ‘straight up information’ (C01) which is honest and accurate to answer their questions and address their uncertainties. This should include experts providing ‘options for moving on’ (C01) with their life and sharing information about amputation, which is tailored around a person's quality of life needs.

#### 3.3.6 Communicate With Respect and Transparency

It was recommended that information should be shared in plain language that can be understood and communicated in a respectful manner to ensure a shared understanding of amputation processes.

#### 3.3.7 Offer Functional Person‐Centred Systems

This category described the streamlined and functional systems, which should be in place to support person‐centred decisions for amputation. People with a DFU valued continuity of staff to ensure all health practitioners are aware of a person's circumstances and medical history.

## 4 Discussion

**FIGURE 3:**
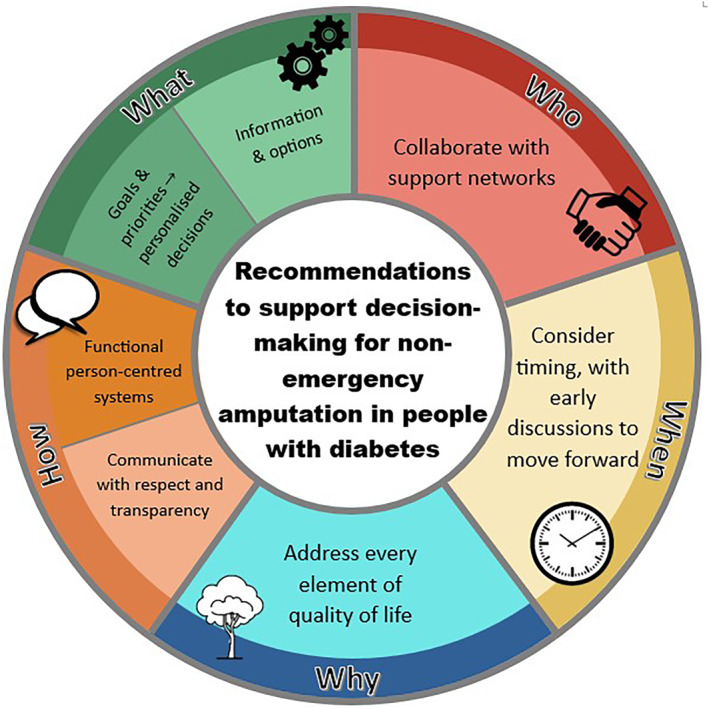
Decision‐making wheel for non‐emergency amputation due to diabetes.

This study is built on previous research to gather and prioritise recommendations that support decision‐making for non‐emergency amputation due to diabetes. There were initially 28 recommendations, which were synthesised to seven categories. To the best of our knowledge, this is the first study to collaborate with and bring together data from published literature, PwLE, family, health practitioners and health experts about the decision‐making process for non‐emergency amputation related to diabetes. This research led to key recommendations about decision‐making including timing, shared involvement, and rationale for amputation‐decisions. As such, this series of recommendations was well aligned to inform a decision‐making tool that provides guidance for the ‘who, what, when, why, and how’ of amputation decision‐making (see Figure 3).

‘Consider timing, with early discussions to move forward’ (‘WHEN’) was weighted as the most important category for decision‐making. Recommendations from both cohorts centred around finding the optimal time to initiate tailored discussions for amputation with general agreement that early discussions were beneficial in supporting people to feel informed about their options. Previous studies have reported conflicting ideas on the ideal timing of conversations with some researchers reporting that people prefer having conversations about amputation upfront [6, 22]. The participants in this research recognised this concept as relevant to them; however, it was acknowledged that timing of an amputation is unique to each individual and their circumstances and needs.

Quality of life was a common idea within the recommendations gathered from both cohorts, providing evidence for ‘WHY’ these decisions for non‐emergency amputation are important. Inconsistencies in the definition of quality of life have been reported in the literature [23]. Some studies describe an individual's subjective wellbeing including their goals, values and expectations as key contributors to quality of life, whilst others focus on objective measurable factors such as pain, mental health, and physical function [23, 24]. Given different perspectives of quality of life, health professionals need to explore the priorities, goals, and what quality of life means to different people. The NGT revealed the value of considering functional, emotional, and financial needs when considering quality of life in amputation decisions. These factors combined can predict a person's outcomes and post‐amputation quality of life [25].

Collaborating with people with a DFU, family members, community, and multi‐disciplinary teams (positioned as ‘WHO’ in the wheel) was recommended when making decisions for amputation. It is known that patient participation in healthcare decision‐making offers benefits such as increased trust, quality of life, and feelings of empowerment and satisfaction [26, 27].

Provision of information (defined as the ‘WHAT’) is needed to make decisions and support a mutual understanding about amputation for people with a DFU and health experts. Establishment of goals are the output of these decisions, which are influenced by their understanding of amputation, as well as personal values. This finding is consistent with existing evidence that goal setting amongst patients improves quality of life through self‐efficacy [28]. Health practitioners, therefore, must facilitate effective goal setting and information sharing amongst people with a DFU, to support informed collaborative decisions. Trust between a patient and their health practitioner facilitated goal setting conversations with people with a DFU [6].

Functional systems and streamlined processes that are person‐centred were also identified (‘HOW’ in the wheel). A person‐centred care approach fosters trust and respect by seeking to understand what is important to people, to work together to make shared decisions [29]. For PwLE and family, person‐centred care was achieved through consistency of staff to ensure practitioners were familiar with their treatment history for trust. This substantiates a previous study with people with a DFU, which described the importance of health practitioner continuity for trust and rapport [4]. A systematic review also demonstrated that continuity of staff supports patients to share information about themselves, enabling management decisions to be tailored to the person's needs [30]. For health experts, person‐centred care was established through taking the time to understand the quality of life needs for people with a DFU. This study is the first to report on the value of person‐centred decision‐making in the context of non‐emergency amputation due to diabetes.

### 4.1 Limitations and Recommendations for Further Research

The online platform may have impacted the flow of group discussions and group dynamics. All PwLE and family members were recruited from the same metropolitan public hospital meaning future research incorporating the perspectives of people living in different geographical regions is recommended. As all PwLE and family members were from a European background, a limitation is the translatability of these findings to other cultural groups including Aboriginal and Torres Strait Islander peoples. Future research with a larger sample from family members and from PwLE of a major amputation would also be beneficial. The first activity of the NGT workshops that required participants to reflect on presented themes may have limited generation of own ideas from the participants.

Further research into how these recommendations can be effectively implemented into multi‐disciplinary clinics is now required. This involves testing of the decision‐making wheel in these settings and collaborating with decision‐makers to ensure the implementation process reflects their needs. Identifying how the recommendations from this study can be implemented into existing clinical practice guidelines will ensure they are user‐friendly, and able to be translated and implemented across clinical settings. The decision aid developed by Dillion et al. (2021) supports people facing a partial foot amputation due to peripheral arterial disease, to decide between different levels of amputation. The prominence of quality of life within this decision aid is also featured in the decision‐making wheel established in this study. Further research is now necessary to understand how the recommendations for amputation decision‐making due to diabetes from this study and the decision aid for amputations due to peripheral arterial disease can work together for implementation in practice.

## 5 Conclusion

The outcomes of this study are the first steps towards establishing evidence‐based guidelines for making decisions for amputation. A person‐centred approach is required when making decisions for non‐emergency amputation. This is achieved by a mutual understanding of goals and priorities, and differing meanings of quality of life through systems that enable continuity of health practitioners. Identifying the optimal time for amputation may be facilitated through early discussions. Further research into how these recommendations can be implemented into multi‐disciplinary clinics across different settings is now required.

## Author Contributions

**Emilee Kim Ming Ong:** conceptualization, investigation, methodology, formal analysis, project administration, visualization, writing – original draft, writing – review and editing. **Carolyn Murray:** conceptualization, formal analysis, supervision, writing – review and editing. **Susan Hillier:** conceptualization, formal analysis, supervision, writing – review and editing. **Ryan Causby:** conceptualization, formal analysis, supervision, writing – review and editing.

## Funding

The principal researcher is a PhD candidate and is receiving a Research Training Programme Scholarship from the University of South Australia.

## Ethics Statement

Ethics approval was obtained from Southern Adelaide Local Health Network (SALHN) (SALHN ethics LNR/23/SAC/85) and the University of South Australia Human Research Ethics Committees (reference number 205750). All participants provided written informed consent to participate in this study.

## Consent

Participants were informed that participation was voluntary and provided written informed consent prior to the nominal group workshops. Participants were reminded that participation in this study or withdrawing at any time would not affect their regular care, nor their relationship with staff of the university or health service.

## Conflicts of Interest

The authors declare no conflicts of interest.

## Permission to Reproduce Material From Other Sources

Figures reproduced from previous publications were published under Creative Commons Attribution licence (CC‐BY). Therefore, no additional permissions from these sources were required.

## Supporting information


Supporting Information S1

Supporting Information S2

Supporting Information S3

Supporting Information S4

## Data Availability

Transcripts will not be made available to maintain confidentiality of all participants.
