# Supplementary material for: Using Nominal Group Technique to Gather Recommendations in the Decision‐Making for Amputation Due to Diabetes
Source: J Foot Ankle Res. 2025 Nov 3;18(4):e70095. doi: 10.1002/jfa2.70095 (PMC12582910; doi:10.1002/jfa2.70095)
Supplement: Supplementary file 1 — Supporting Information S1 [file JFA2-18-e70095-s003.docx]

Decision-making for amputation in diabetes

Workshop 28th November 2024

Contents

[Welcome 2](#_Toc177131385)

[Demographic information about you 3](#_Toc177131386)

[Workshop structure 4](#_Toc177131387)

[Workshop aims and objectives 5](#_Toc177131388)

[Part 1: Summary of previous study findings 6](#_Toc177131389)

[Study 1 Scoping review 6](#_Toc177131390)

[Study 2 Interviews with people with ulcers/ amputations, health practitioners, and expert stakeholders 7](#_Toc177131391)

[Part 2: Discussion of themes describing decision-making processes 8](#_Toc177131392)

[Part 3: Recommendation generation and voting 9](#_Toc177131393)

[Extra writing space 10](#_Toc177131394)

# Welcome

Thank you for your interest in participating in our workshop to establish recommendations for amputation in people living with diabetes-related foot ulcers (DFU). This information booklet includes a summary of the research findings from the previous studies which have been completed as part of this PhD project, please take the time to read through this information.

Please ensure you have read through the participant information sheet and returned the consent form at the start of the workshop, feel free to get in touch if you require any assistance. Please note that the discussions during the workshop will be audio recorded for data analysis, please let me know if you do not wish for the session to be recorded.

Finally, please do not hesitate to contact me if you have any further questions or concerns. Thank you once again for your time and participation in our workshop today.

Emilee Ong
*PhD Candidate*

Dr Ryan Causby, Dr Carolyn Murray, and Prof. Susan Hillier

*Research supervisors*

University of South Australia

# Demographic information about you

| **Full name** |  |
| --- | --- |
| **Postcode** |  |
| **Age** |  |
| **Gender** |  |
| **Employment status/ occupation** |  |
| **Living arrangements (i.e.. Partner, alone, carer etc)** |  |
| **Location of ulcer on the foot** |  |
| **Approximate duration of ulcer** |  |
| **Have you had a previous ulcer? (yes/no)** |  |
| **History of amputation (yes (site)/no)** |  |

# Workshop structure

**Start time: approx. 10.30am**

| Topic | Description | Duration |
| --- | --- | --- |
| Welcome | Introduction and workshop aims. Presentation of previous research findings. | 15min |
| Discussion of themes | Individual reflection on key themes and group discussion. | 15min |
| Recommendation generation | Individual generation of recommendations. | 10min |
| Break | | 5min |
| Voting on recommendations | Sharing of recommendations and group discussion. Voting on recommendations. | 30min |
| Summary of final recommendations | Discussion of final recommendations. | 15min |
| Finish | | |

# Workshop aims and objectives

Today’s workshop forms part of the final study of a PhD project exploring the decision-making processes for lower extremity amputation in people with diabetes.

**PhD aim**

PhD Aim

The aim of this PhD Project is to discuss the way decisions should be made when someone might need an amputation.

We want to understand the views and opinions of people who might need the amputation, as well as the clinicians and practitioners involved. From this we will create a set of recommendations to help everyone make this decision.

**Workshop aims/objectives**

Workshop Aim

The aim of this workshop is to use your experience as someone who has had to think about amputation. Then together we will write some potential recommendations for people to use in the future to help this process and make decisions in the best way possible.

Workshop Objectives

- To think about our research findings in the context of your own experience to create recommendations for the process of making decisions about amputation.
- To vote on the most important recommendation/s made by the group.

# Part 1: Summary of previous study findings

## Study 1 Scoping review- A review of existing research

**Decision-making processes for non-emergency diabetes-related lower extremity amputations: A scoping review**

- We reviewed the research that has already been conducted to understand the clinical reasoning considerations (the factors clinical staff have to consider) and decision-making processes (how decisions are made) for lower limb amputation in people who have a diabetes-related foot ulcer.
- Majority of the current research focuses on the person’s health conditions and their wound/s to inform decisions for amputation.
- Very few research articles considered how an amputation may impact a person’s function and activity levels after the surgery.
- Personal and lifestyle factors were important considerations for amputation.


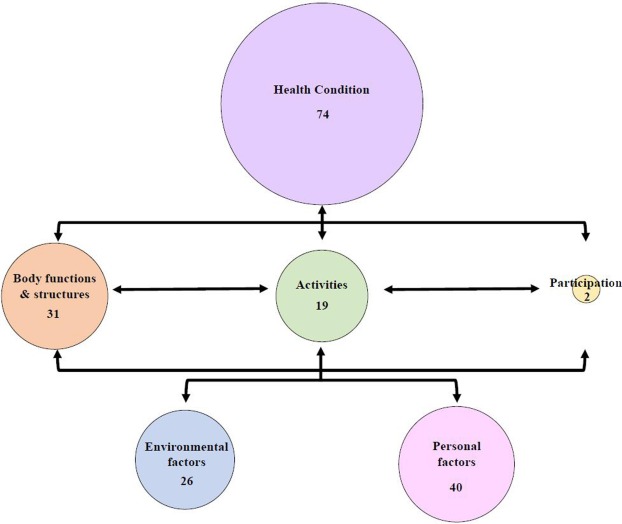


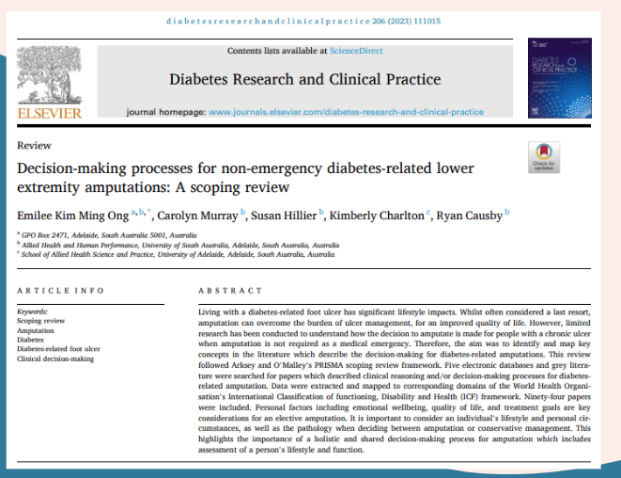


Link to the article: <https://www.sciencedirect.com/science/article/pii/S0168822723007787?via%3Dihub#f0010>

## Study 2 Interviews with people with ulcers/ amputations, health practitioners, and expert stakeholders

**‘Leaving the door open’: Perspectives of decision-making for non-emergency diabetes-related amputation**

- One-to-one interviews were conducted with 26 people (13 female and 13 male)
- Participants included nine people with a diabetes-related foot ulcer or amputation, nine health practitioners, and eight researchers, located across five countries.
- The findings showed four main themes to consider when making the decision to amputate or not (see figure below).
- Work commitments, function and lifestyle impacts from amputation, presence of support networks, and wound features (like severity, position, size etc) were all identified as important considerations when making the decision to amputate.
- Living with a diabetes-related foot ulcer presented daily challenges which pushed people to a tipping point. This is when amputation is considered to be the best solution to manage these challenges and enable people to move onto the next chapter of their life.

# Part 2: Discussion of themes describing decision-making processes

**Activity**: Have a read through the diagram below. Please pick two of the ideas (themes) in the boxes below which you relate the most with in your decision-making for amputation due to diabetes (5 minutes). We will then discuss your choices as a group (10 minutes).

Notes:

# Part 3: Recommendation generation and voting

**Question: Looking at the themes below, what are your key recommendations to support people like yourself who are making decisions for amputation?**

Initial notes:

**Please write your final recommendations on the separate pieces of paper provided to you.*

# Extra writing space
