# Supplementary material for: Using Nominal Group Technique to Gather Recommendations in the Decision‐Making for Amputation Due to Diabetes
Source: J Foot Ankle Res. 2025 Nov 3;18(4):e70095. doi: 10.1002/jfa2.70095 (PMC12582910; doi:10.1002/jfa2.70095)
Supplement: Supplementary file 2 — Supporting Information S2 [file JFA2-18-e70095-s004.docx]

Practitioner and stakeholder consultation: decision-making considerations for non-emergency amputation in diabetes

Co-design workshop 1st August 2024

Contents

[Welcome 2](#_Toc172537125)

[Workshop structure 3](#_Toc172537126)

[Workshop aims and objectives 4](#_Toc172537127)

[Part 1: Summary of previous study findings 5](#_Toc172537128)

[Study 1 Scoping review 5](#_Toc172537129)

[Study 2 Interviews with people with ulcers/ amputations, health practitioners, and expert stakeholders 6](#_Toc172537130)

[Part 2: Discussion of themes describing decision-making processes 7](#_Toc172537131)

[Part 3: Recommendation generation and voting 8](#_Toc172537132)

[Extra writing space 10](#_Toc172537133)

# Welcome

Thank you for your interest in participating in our co-design workshop to establish recommendations for non-emergency amputation in people living with diabetes-related foot ulcers (DFU). This information booklet includes a summary of the research findings from the previous studies which have been completed as part of this PhD project. Please take the time to read through this information which includes the main question to be discussed on the day in preparation for the workshop.

Please ensure you have read through the participant information sheet and returned the consent form prior to the workshop, feel free to get in touch if you require any assistance. Please note that the discussions during the workshop will be audio recorded for data analysis, please let me know if you do not wish for the session to be recorded.

Finally, please do not hesitate to contact me if you have any further questions or concerns. We look forward to seeing you online on 1^st^ August 2024 at 9am (Adelaide-Australian Central Standard Time).

Emilee Ong
*PhD Candidate*

Dr Ryan Causby, Dr Carolyn Murray, and Prof. Susan Hillier

*Research supervisors*

University of South Australia

# Workshop structure

Location: Zoom (please use the Zoom link which has been emailed to you).

| Time | Topic | Description | Duration |
| --- | --- | --- | --- |
| 9:00-9:15am | Welcome | Introduction and workshop aims. Presentation of previous key findings. | 15min |
| 9:15-9:30am | Discussion of themes | Individual reflection on key themes and group discussion. | 15min |
| 9:30-10:00am | Recommendation generation | Individual generation of recommendations. Sharing of recommendations and group discussion. | 30min |
| Break | | | 5min |
| 10:05-10:15am | Voting on recommendations | Voting on recommendations. Final summary of votes. | 10min |
| 10:15-10:30am | Summary of final recommendations | Discussion of final recommendations. | 15min |
| Finish | | | |

# Workshop aims and objectives

Today’s workshop forms part of the final study of a PhD project exploring the decision-making processes for lower extremity amputation in people with diabetes.

**PhD aims and objectives**

PhD Aim

The aim of this PhD project is to explore decision-making about lower extremity amputation in the care of people with diabetes-related foot ulcers from the perspectives of the people themselves, practitioners, and experts, to inform a series of recommendations for clinical practice and increase the body of knowledge about diabetes-related amputations.

PhD Objectives

- To review and summarise the available information about clinical decision-making processes about lower extremity amputation for people living with a chronic diabetes-related foot ulcer. (Study 1)
- To explore patient, health practitioner, and expert stakeholder experiences of decision-making in the management of people with a diabetes-related foot ulcer who may require an amputation. (Study 2)
- To develop a series of recommendations for people involved in decision-making for non-emergency amputation due to a diabetes-related foot ulcer, and to evaluate these recommendations using patient, practitioner, and expert stakeholder consultation. (Study 3)

**Workshop aims/objectives**

Workshop Aim

The aim of this workshop is to incorporate your expertise and experience as a practitioner and/or expert in your field to collaboratively establish a series of recommendations that could be used to support people living with diabetes and health practitioners involved in making decisions for non-emergency lower extremity amputation in the future.

Workshop Objectives

- To use the findings from previous studies presented today along with your own ideas and expertise, to create and evaluate recommendations to inform clinical guidelines for decision-making in non-emergency amputation due to a diabetes-related foot ulcer.

# Part 1: Summary of previous study findings

## Study 1 Scoping review

**Decision-making processes for non-emergency diabetes-related lower extremity amputations: A scoping review**

- Review question was ‘*what are the clinical reasoning considerations and decision-making processes for lower extremity amputation in people with a DFU?’*
- Five themes were established from the data extracted and each theme was mapped to the World Health Organisation International Classification of Functioning, Disability and Health framework.
- Personal and lifestyle factors were important considerations for amputation.
- A biopsychosocial approach was used to decide if amputation would improve quality of life.


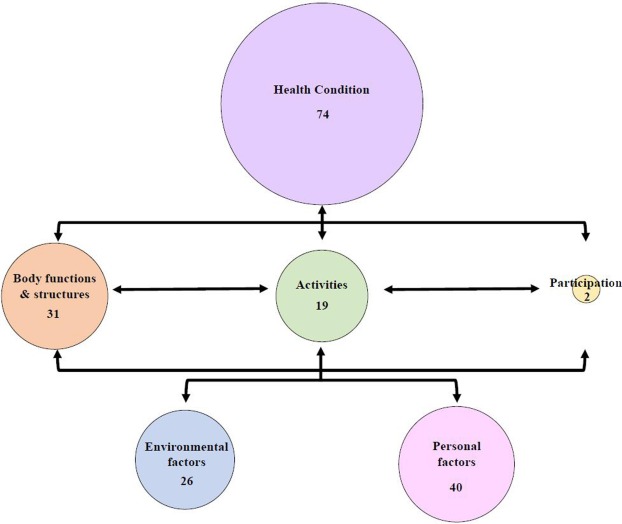


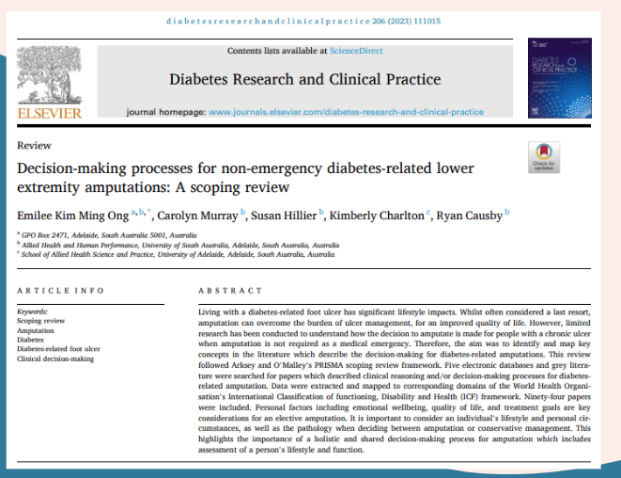


Link to the article: <https://www.sciencedirect.com/science/article/pii/S0168822723007787?via%3Dihub#f0010>

## Study 2 Interviews with people with ulcers/ amputations, health practitioners, and expert stakeholders

**‘Leaving the door open’: Perspectives of decision-making for non-emergency diabetes-related amputation**

- Research question was ‘*what are the perspectives of people with DFU, health practitioners, and experts about the decision-making and experience of lower extremity amputation?’*
- One-to-one semi-structured interviews used for data collection.
- 26 participants recruited including nine people with a diabetes-related foot ulcer or amputation, nine health practitioners, and eight experts, located across five countries.
- There were 13 female and 13 male participants.
- Four themes described the decision-making considerations for amputation (see figure below).
- Work commitments, functional and lifestyle impacts from amputation, presence of support networks, and clinical wound features formed the evidence in a decision for amputation.
- Understanding quality of life needs ensured that decisions for amputation addressed expectations and lifestyle needs.
- Living with a diabetes-related foot ulcer presented daily challenges which pushed people to a tipping point, where amputation was considered to overcome these hardships and enable them to move onto the next chapter of their life.
- Further research is required to understand how person-centred factors can be better incorporated alongside objective clinical assessments in decisions for amputation.

# Part 2: Discussion of themes describing decision-making processes

**Activity**: Have a read through the four key themes and subthemes below. Please circle two of the themes or subthemes which resonate most with you in your decision-making for non-emergency amputation due to diabetes (5 minutes). We will then discuss your selections as a group (10 minutes).

Notes:

# Part 3: Recommendation generation and voting

**Question: With consideration to the themes presented, what are your key recommendations (maximum of three) for practitioners to consider to inform decisions for non-emergency lower extremity amputation in people with a diabetes-related foot ulcer?**

Initial notes:

***Final recommendations:***

**Recommendation 1:**

**Recommendation 3:**

**Recommendation 2:**

# Extra writing space
