# Supplementary material for: Using Nominal Group Technique to Gather Recommendations in the Decision‐Making for Amputation Due to Diabetes
Source: J Foot Ankle Res. 2025 Nov 3;18(4):e70095. doi: 10.1002/jfa2.70095 (PMC12582910; doi:10.1002/jfa2.70095)
Supplement: Supplementary file 3 — Supporting Information S3 [file JFA2-18-e70095-s001.pptx]

## Slide 1
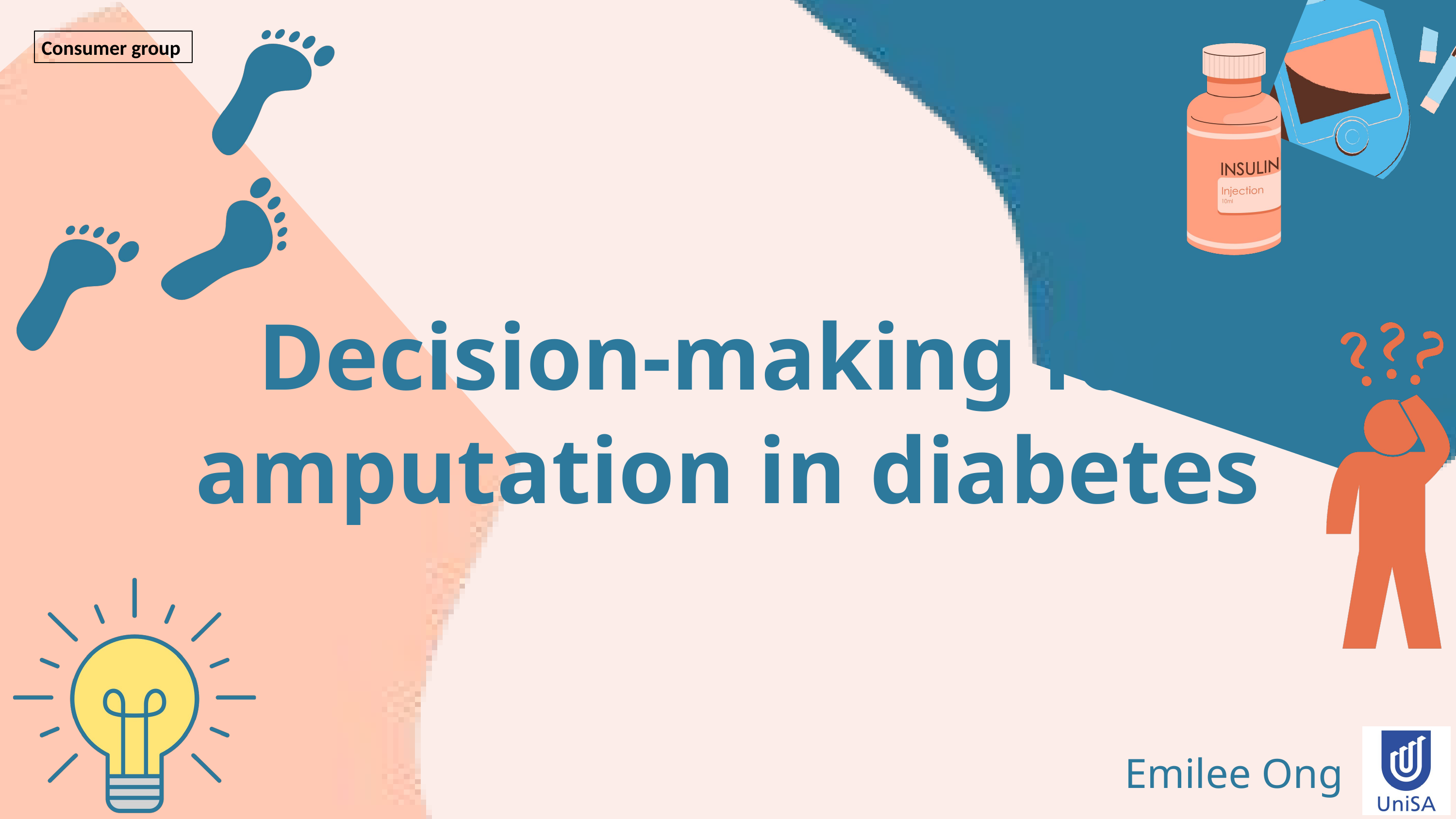

Consumer group
Decision-making for amputation in diabetes
Emilee Ong

## Slide 2
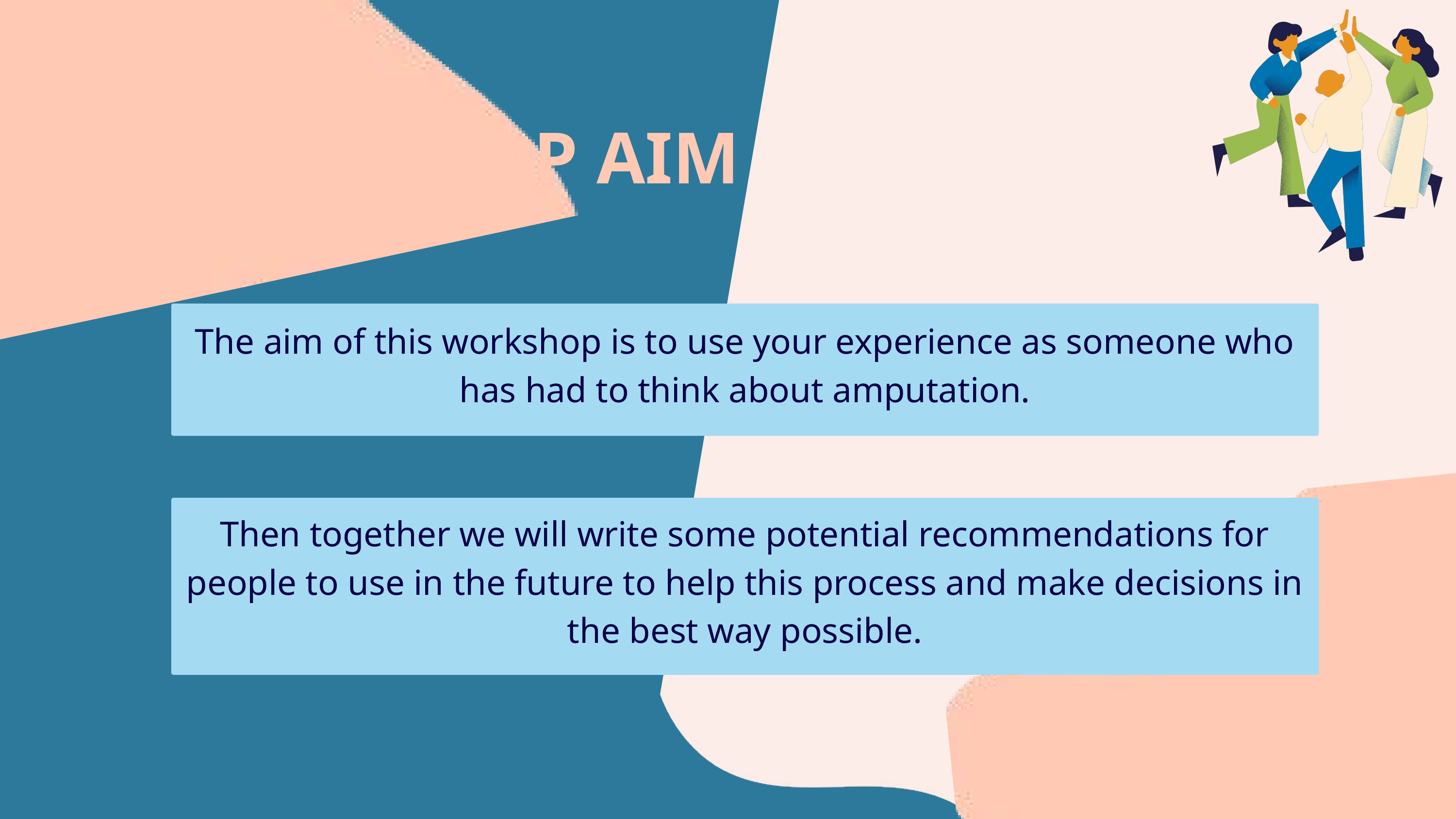

WORKSHOP AIM
The aim of this workshop is to use your experience as someone who has had to think about amputation.
Then together we will write some potential recommendations for people to use in the future to help this process and make decisions in the best way possible.

## Slide 3
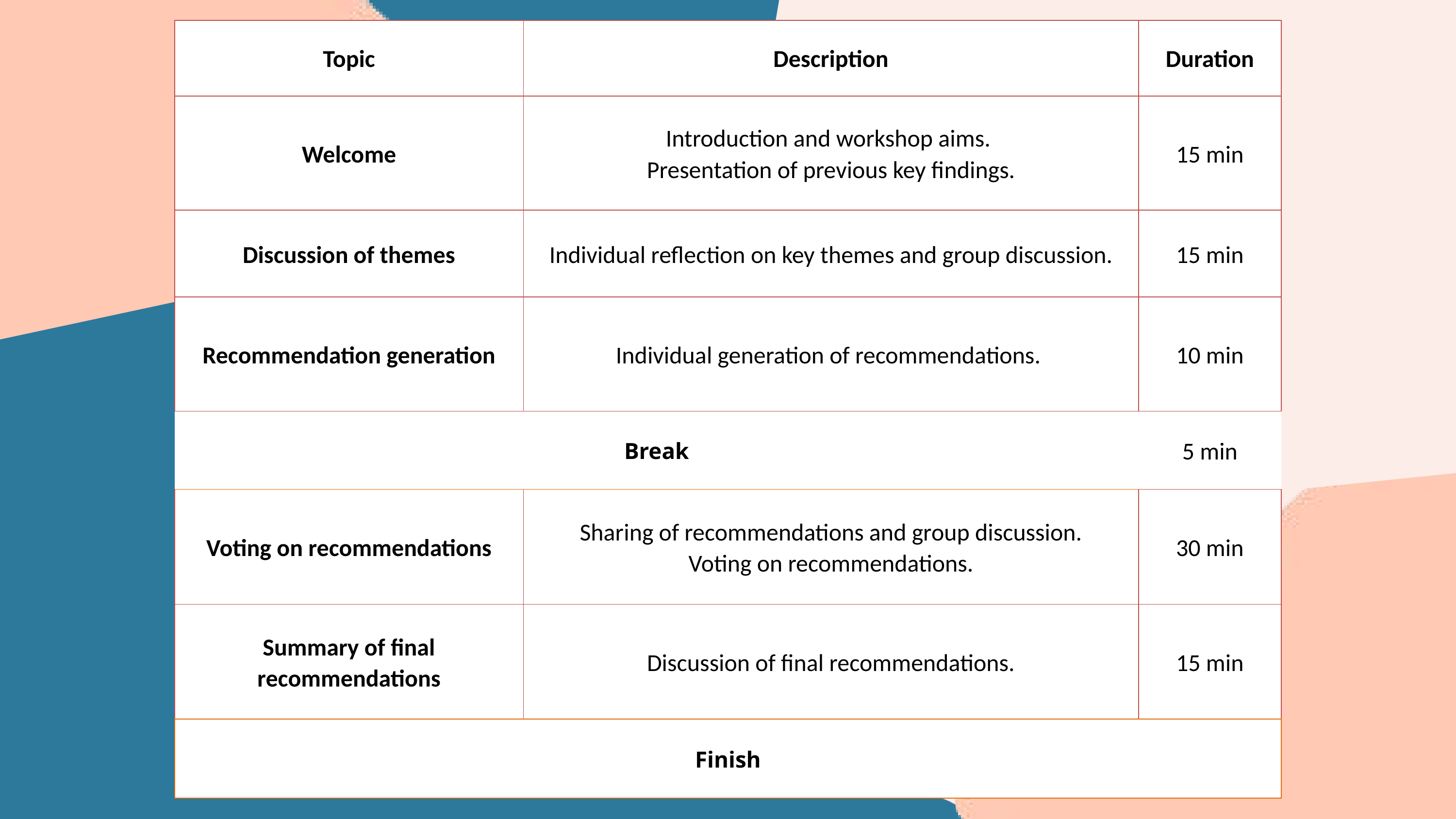

| Topic | Description | Duration |
| --- | --- | --- |
| Welcome | Introduction and workshop aims. Presentation of previous key findings. | 15 min |
| Discussion of themes | Individual reflection on key themes and group discussion. | 15 min |
| Recommendation generation | Individual generation of recommendations. | 10 min |
| Break | | 5 min |
| Voting on recommendations | Sharing of recommendations and group discussion. Voting on recommendations. | 30 min |
| Summary of final recommendations | Discussion of final recommendations. | 15 min |
| Finish | | Finish |

## Slide 4
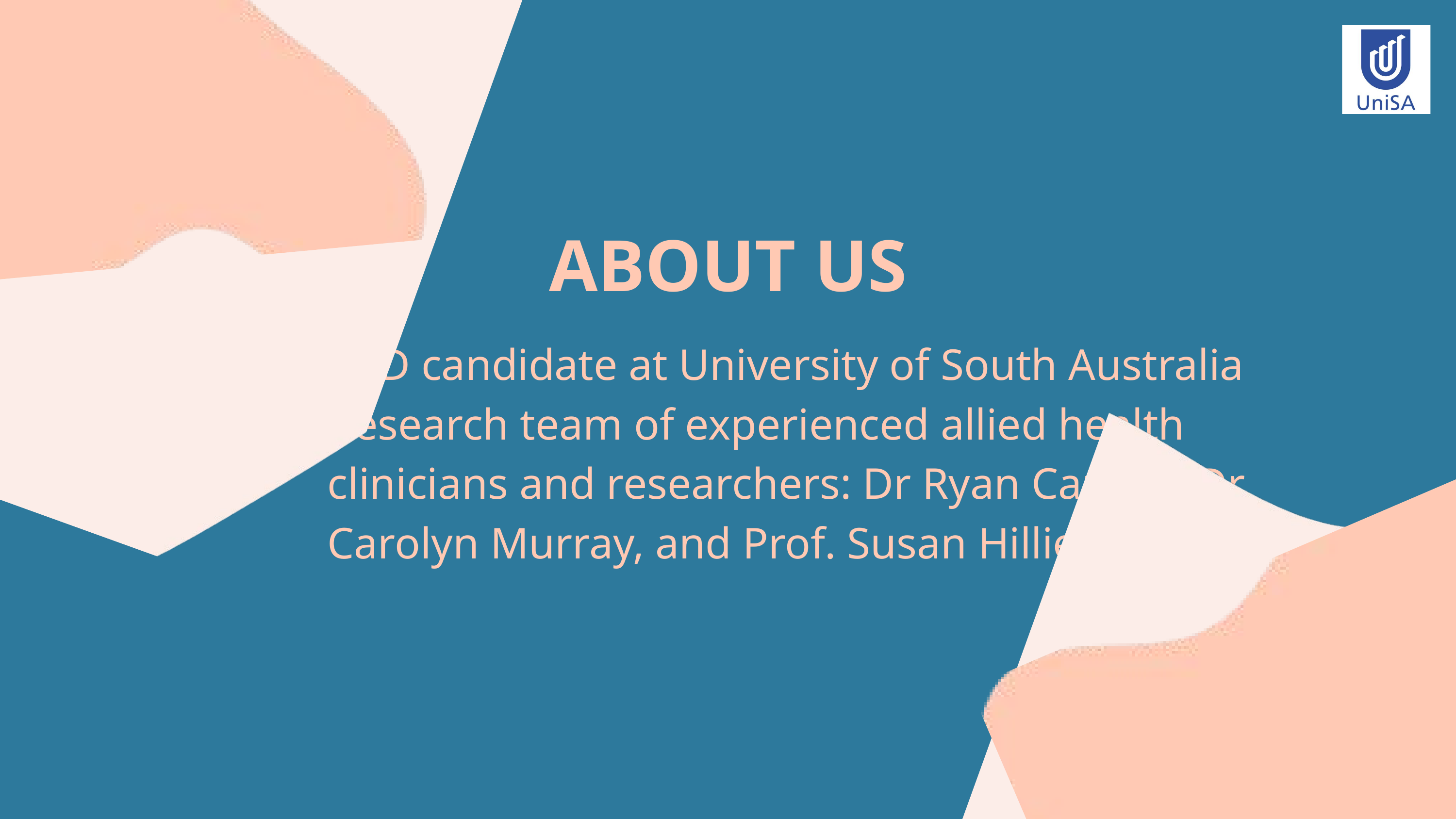

ABOUT US
PhD candidate at University of South Australia
Research team of experienced allied health clinicians and researchers: Dr Ryan Causby, Dr Carolyn Murray, and Prof. Susan Hillier

## Slide 5
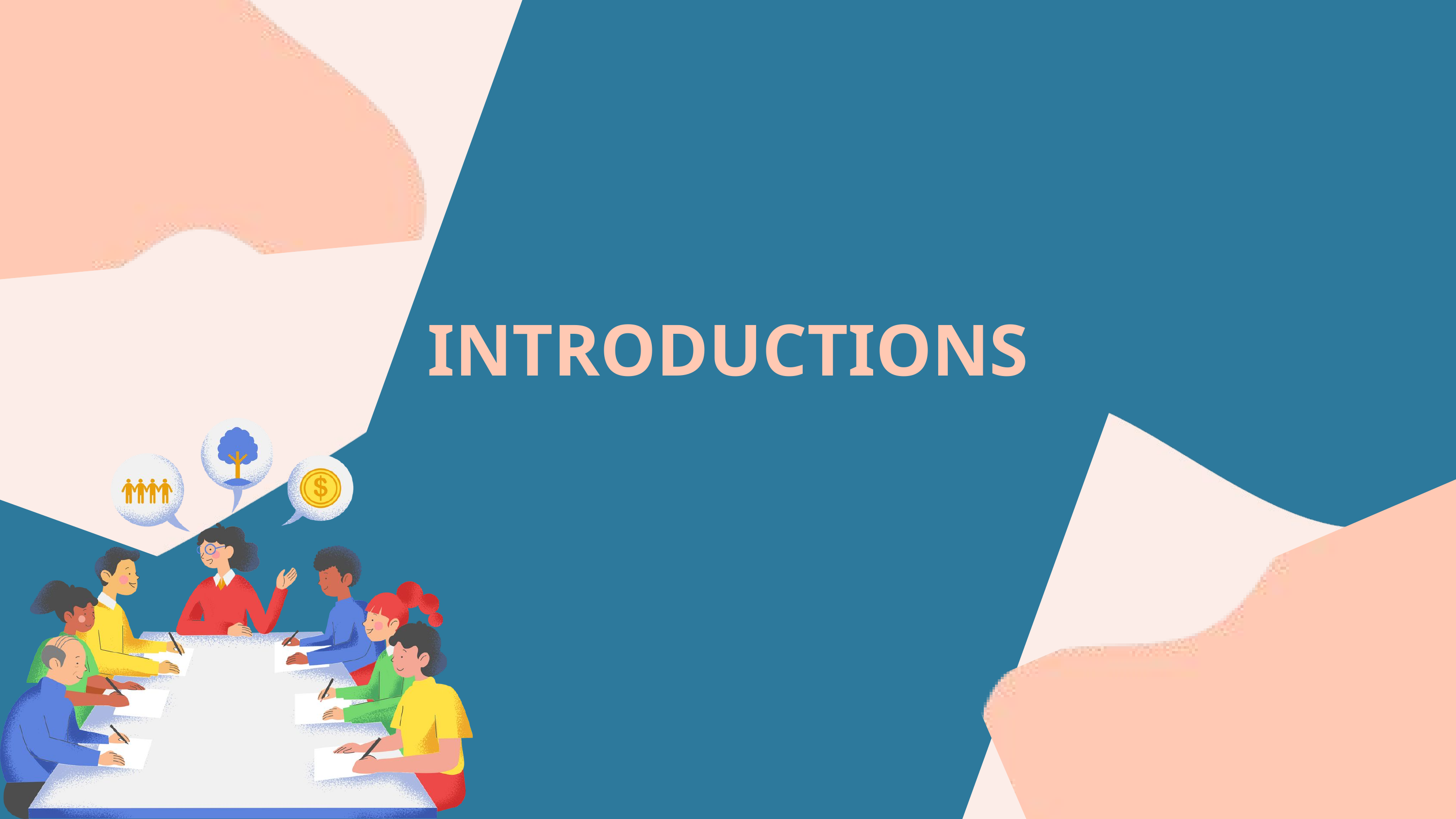

INTRODUCTIONS

## Slide 6
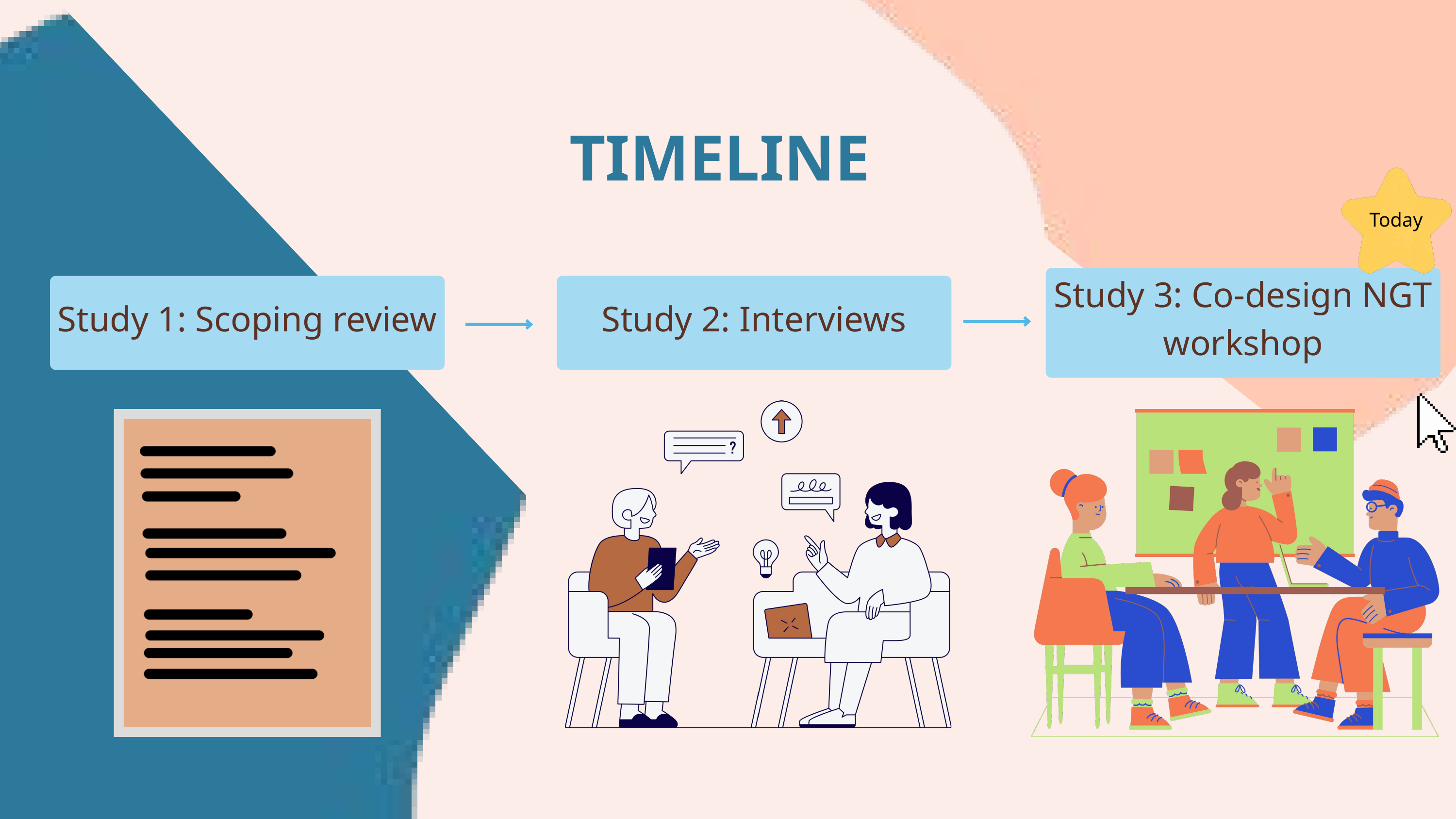

TIMELINE
Today
Study 3: Co-design NGT workshop
Study 1: Scoping review
Study 2: Interviews

## Slide 7
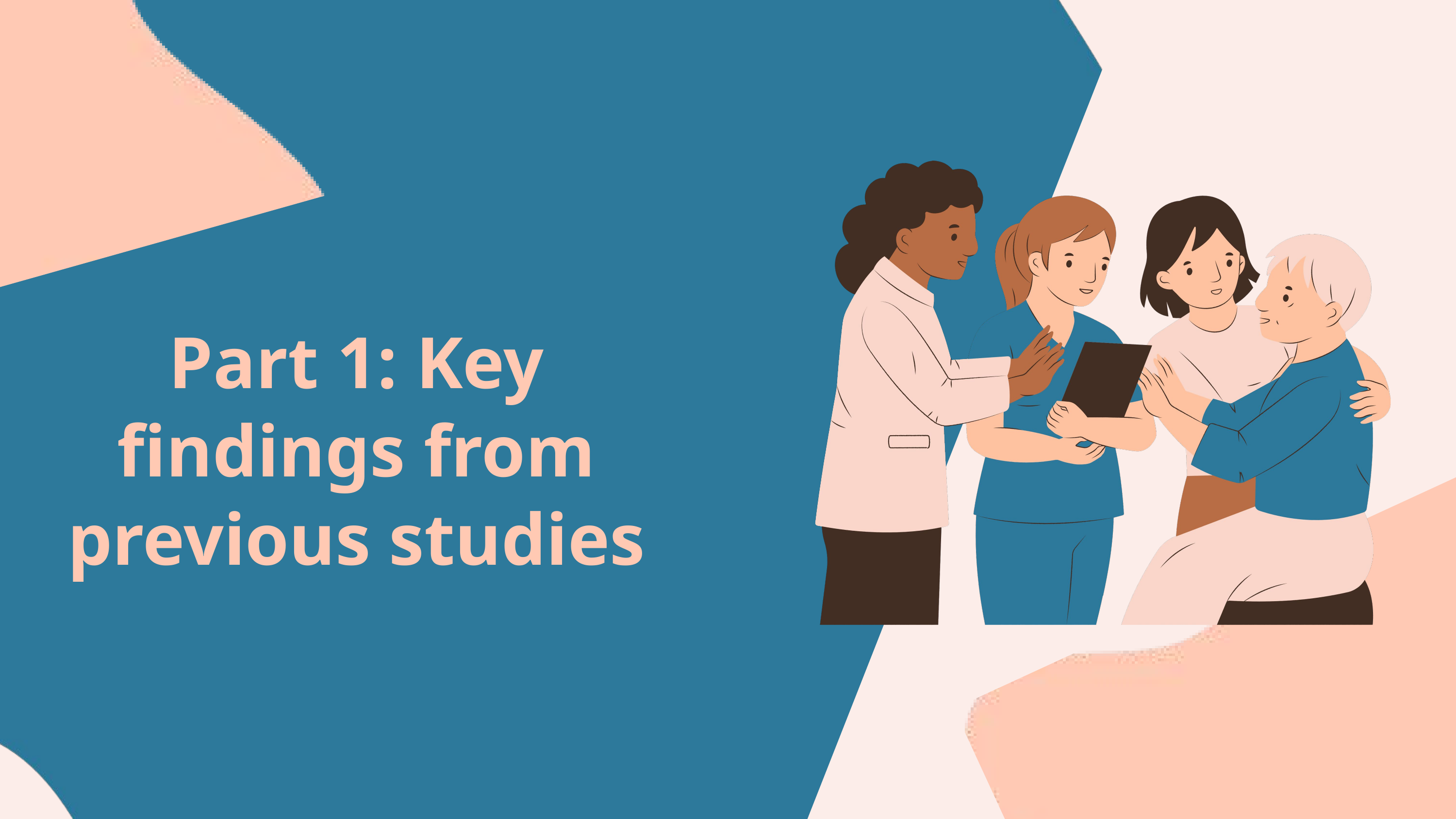

Part 1: Key findings from previous studies

## Slide 8
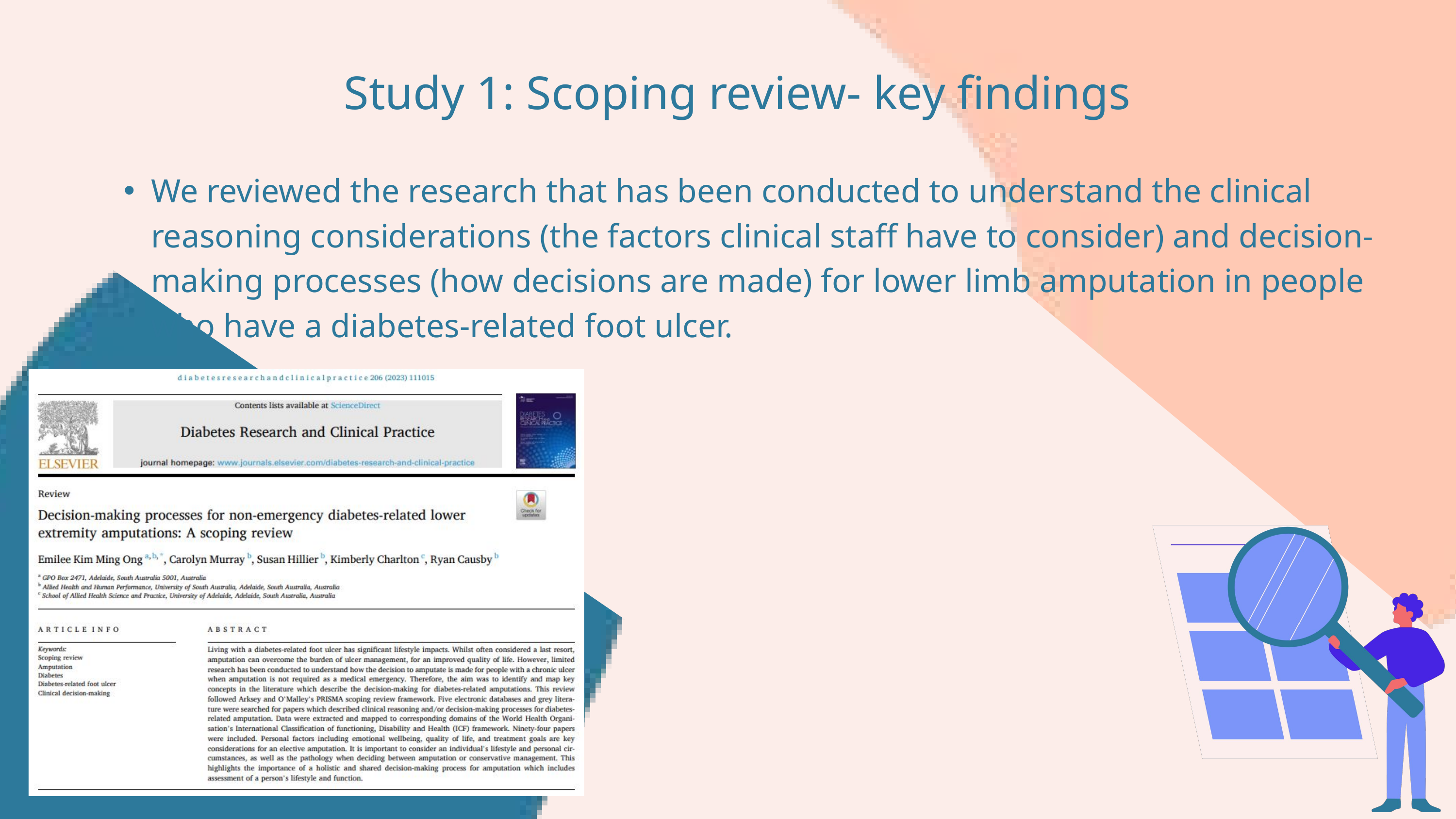

Study 1: Scoping review- key findings
We reviewed the research that has been conducted to understand the clinical reasoning considerations (the factors clinical staff have to consider) and decision-making processes (how decisions are made) for lower limb amputation in people who have a diabetes-related foot ulcer.

## Slide 9
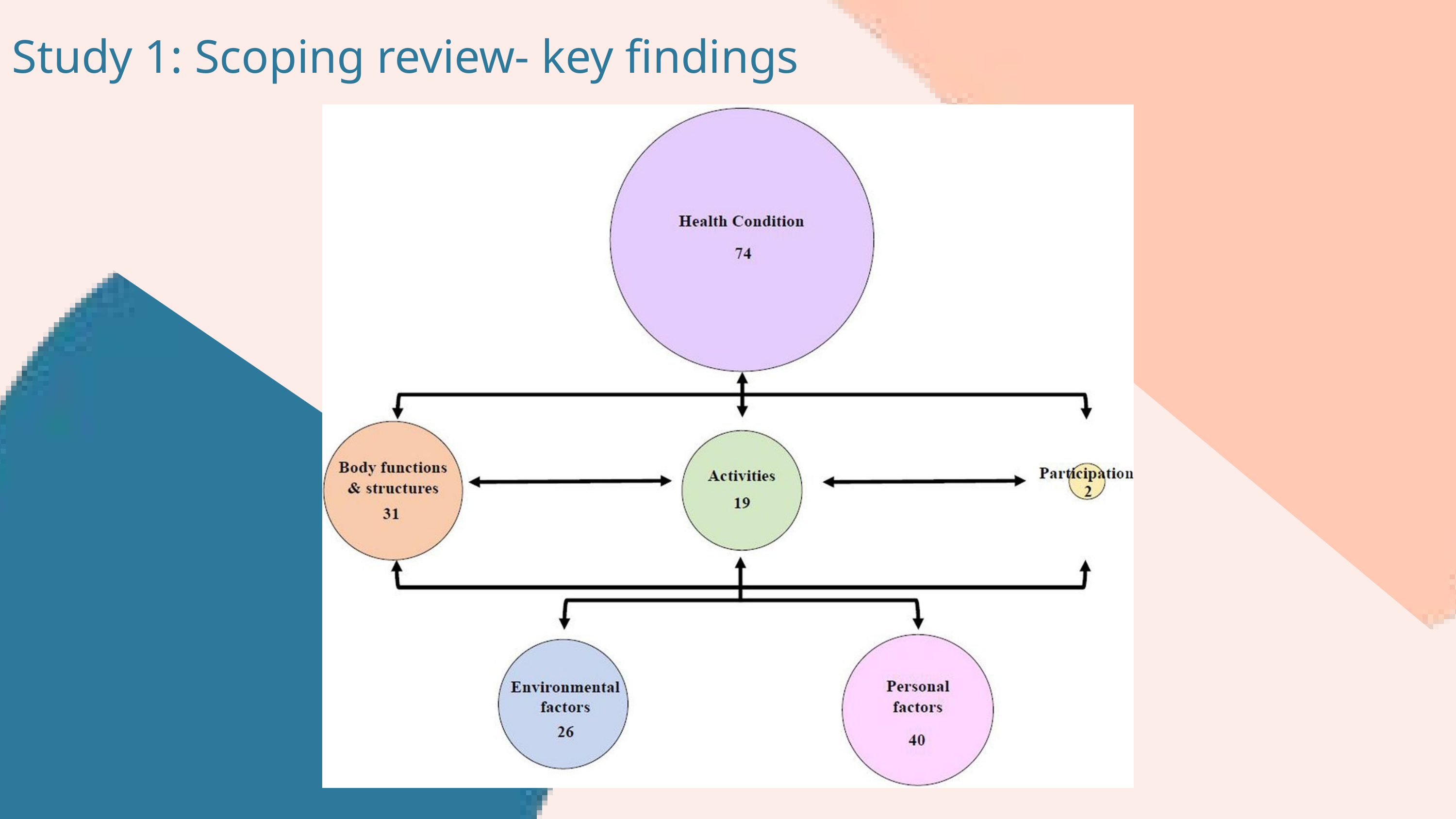

Study 1: Scoping review- key findings

## Slide 10
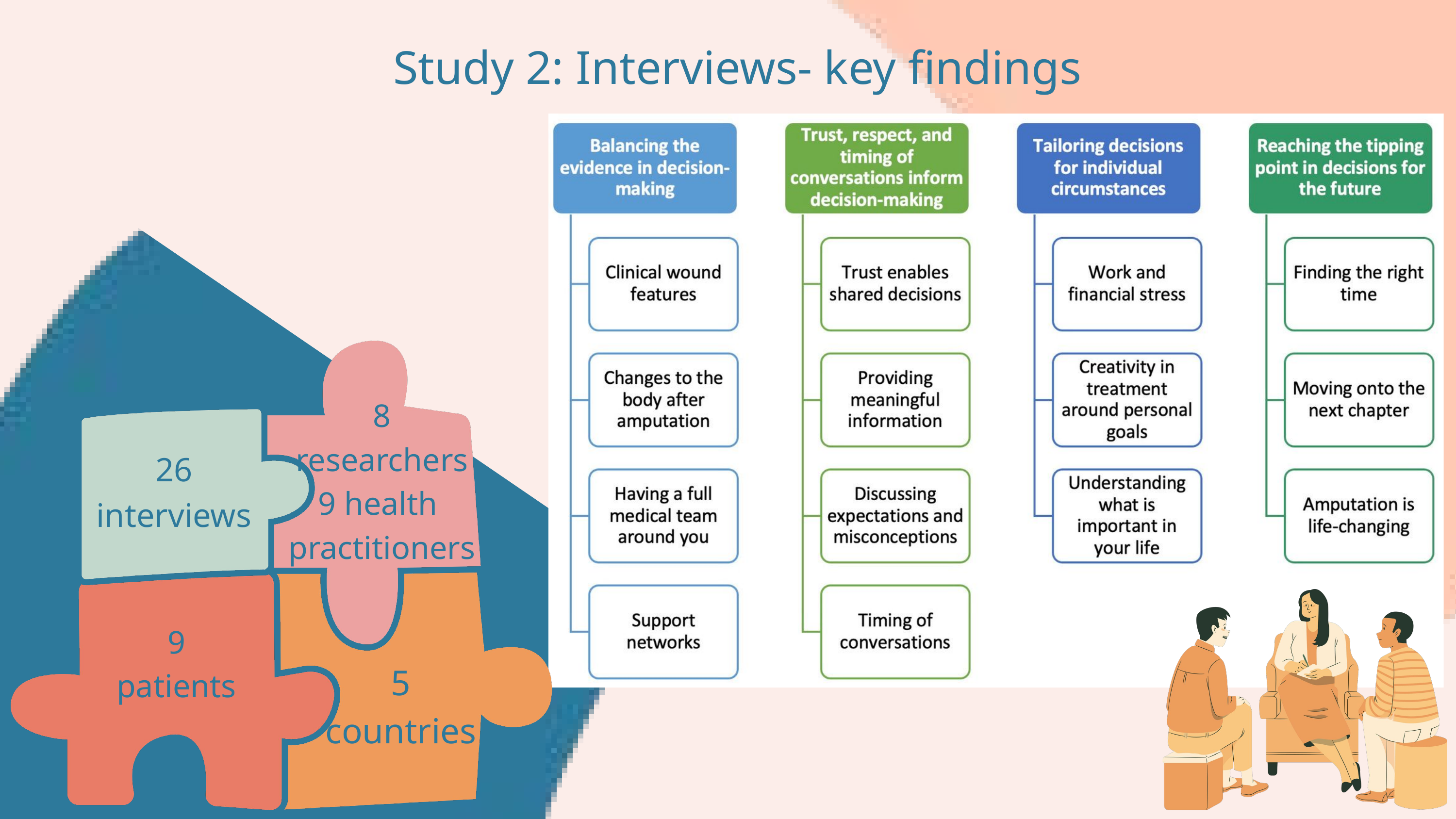

Study 2: Interviews- key findings
8 researchers
9 health
practitioners
26 interviews
9 patients
5 countries

## Slide 11
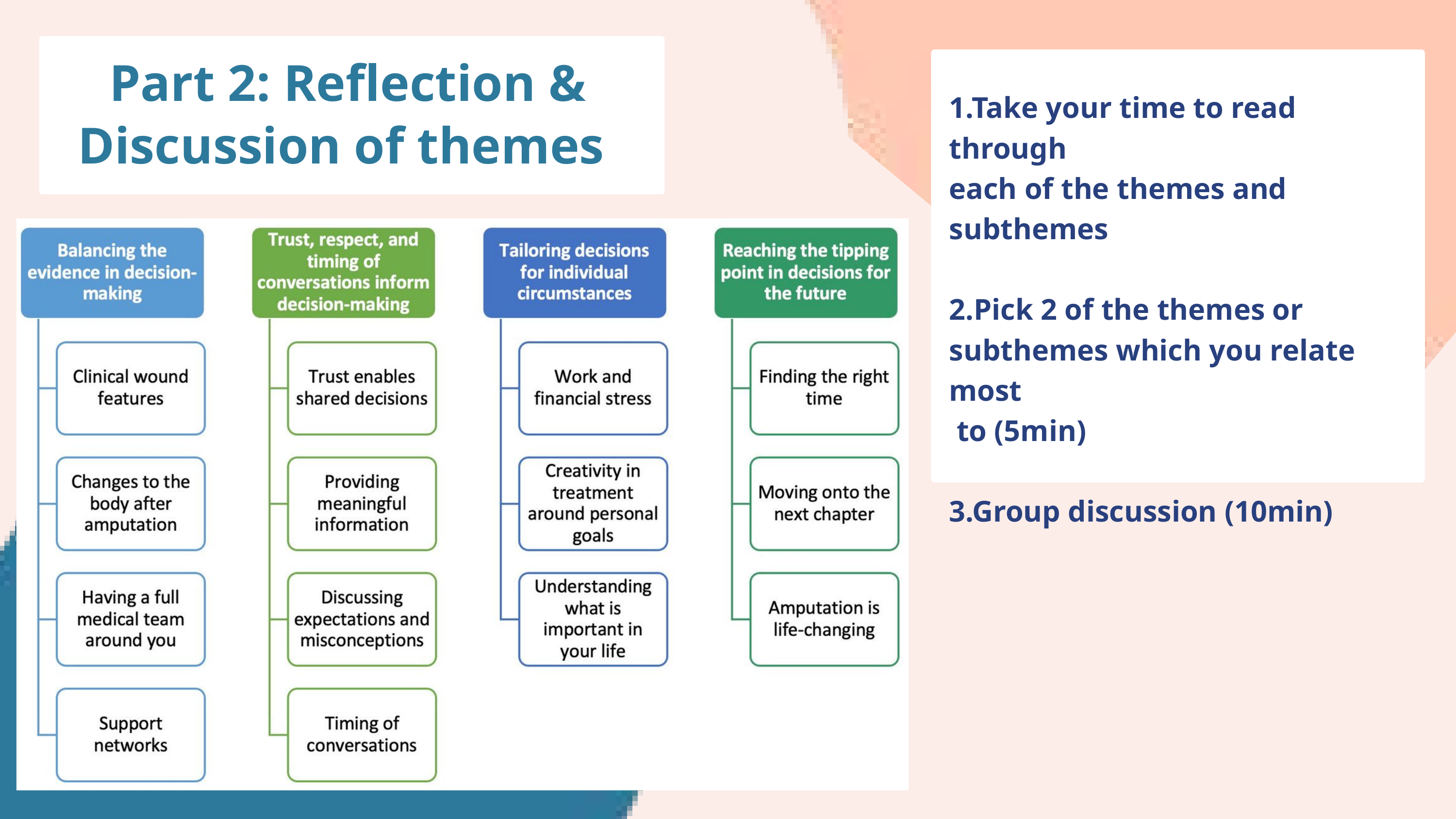

Part 2: Reflection & Discussion of themes
1.Take your time to read through
each of the themes and subthemes
2.Pick 2 of the themes or
subthemes which you relate most
 to (5min)
3.Group discussion (10min)

## Slide 12
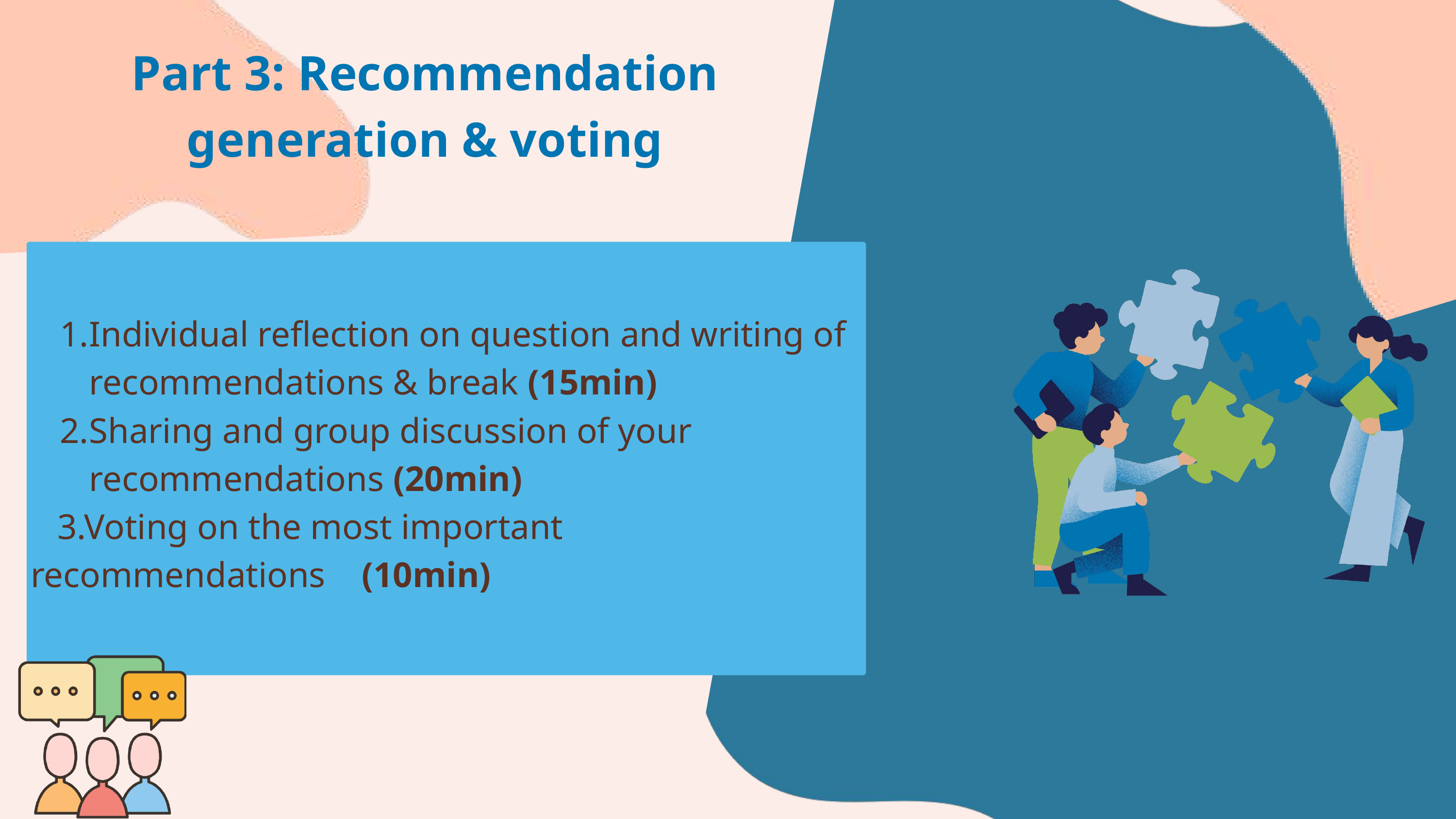

Part 3: Recommendation generation & voting
Individual reflection on question and writing of recommendations & break (15min)
Sharing and group discussion of your recommendations (20min)
 3.Voting on the most important recommendations (10min)

## Slide 13
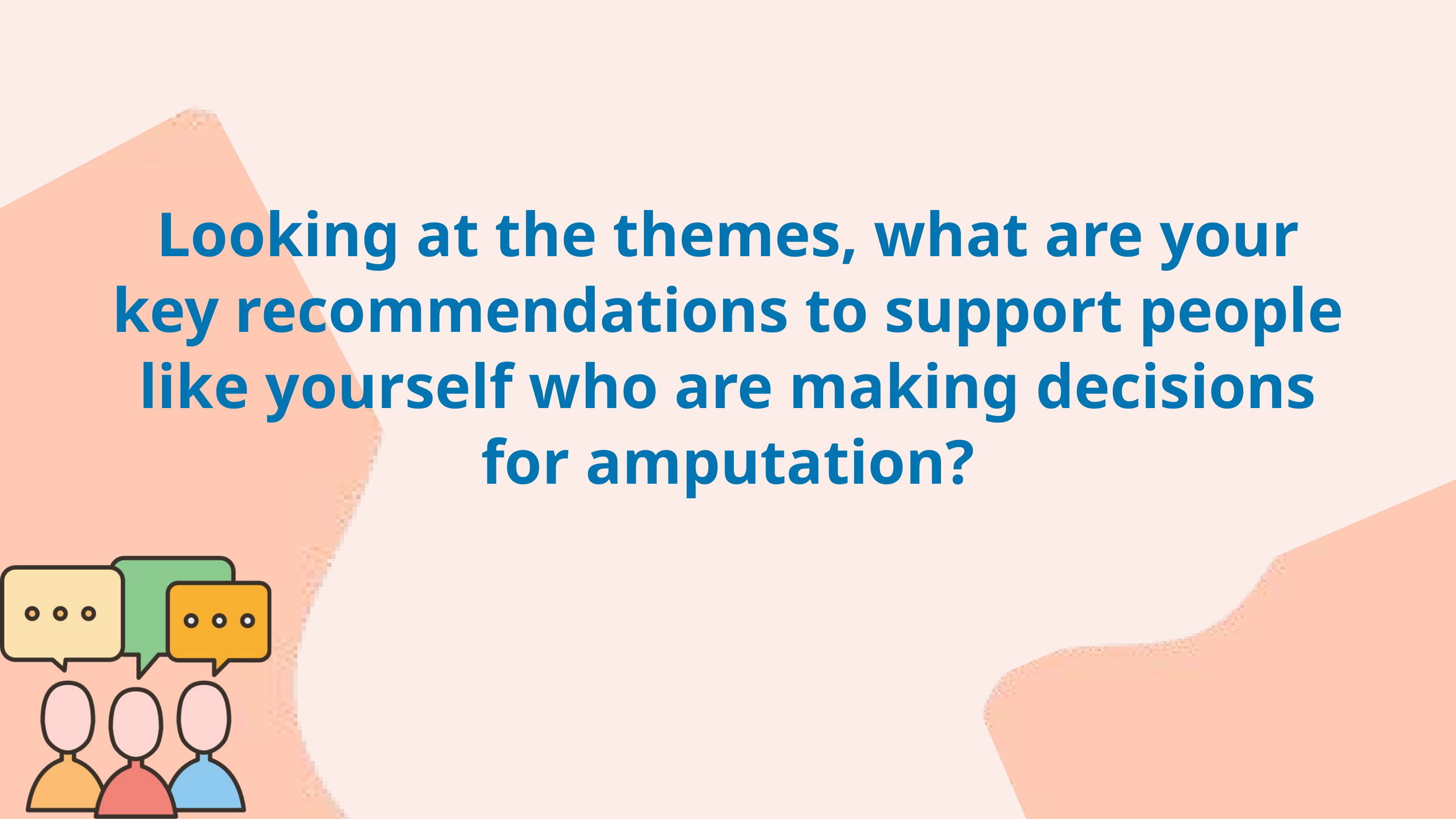

Looking at the themes, what are your key recommendations to support people like yourself who are making decisions for amputation?

## Slide 14
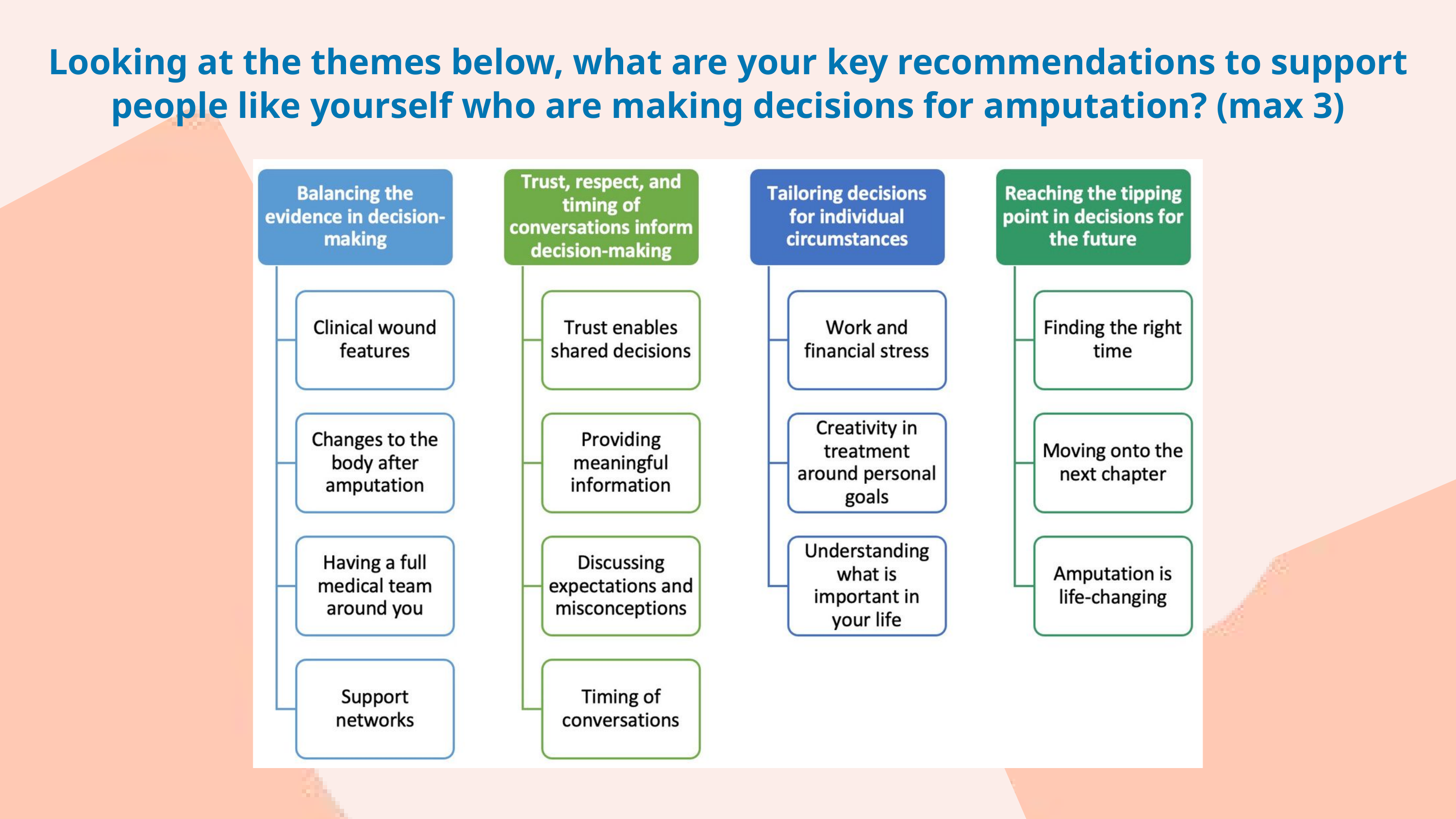

Looking at the themes below, what are your key recommendations to support people like yourself who are making decisions for amputation? (max 3)

## Slide 15
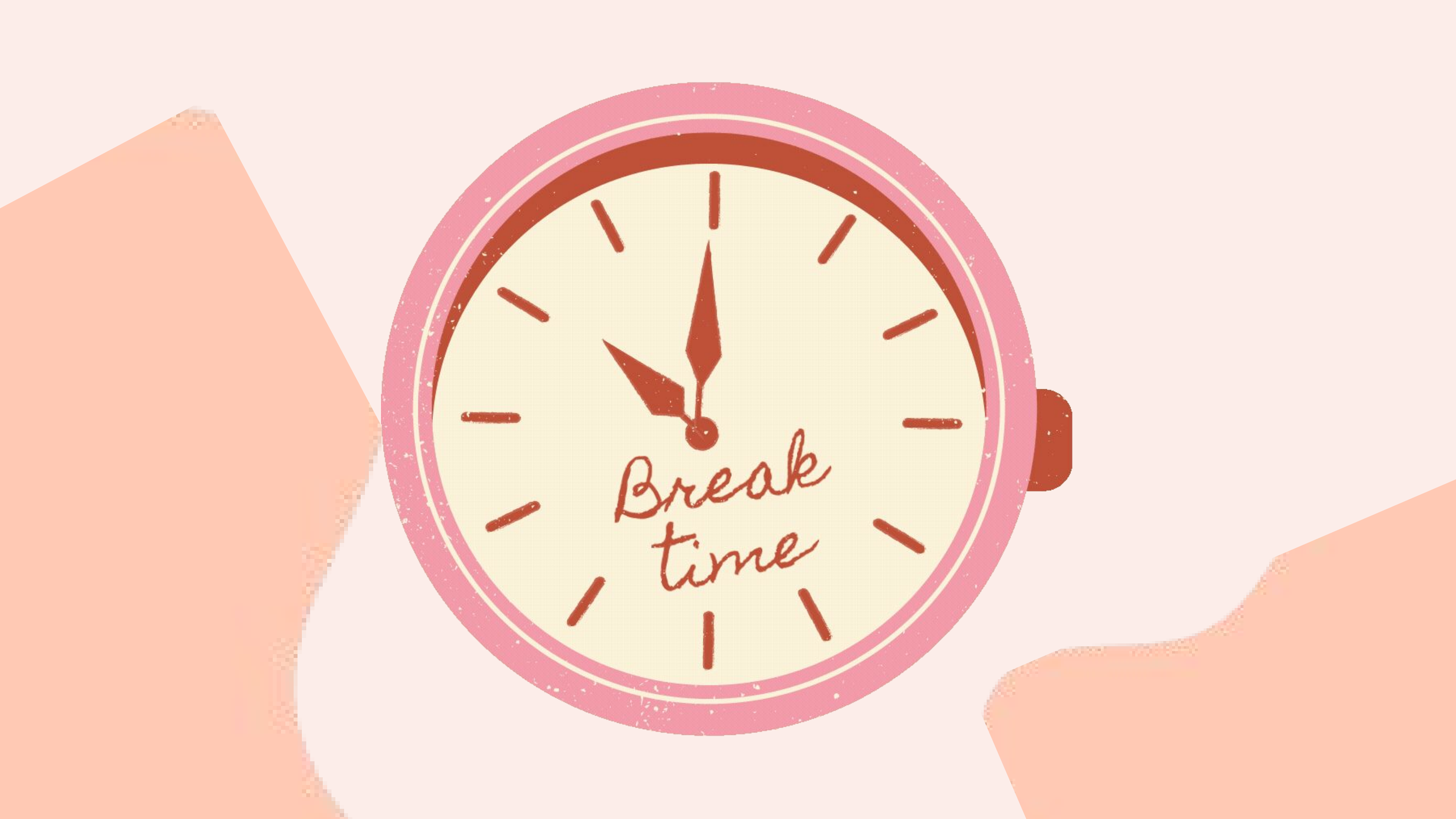

## Slide 16
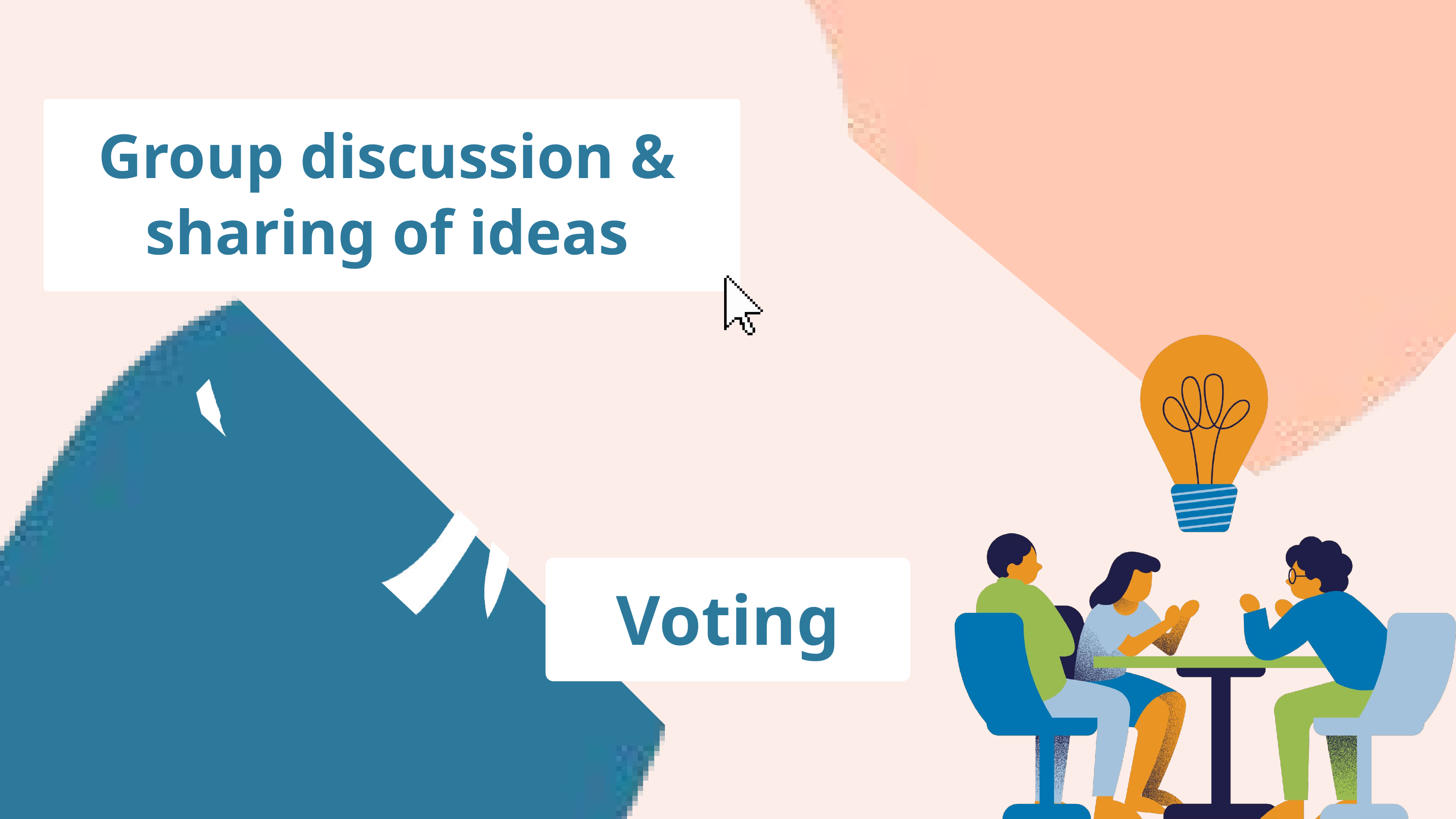

Group discussion & sharing of ideas
Voting

## Slide 17
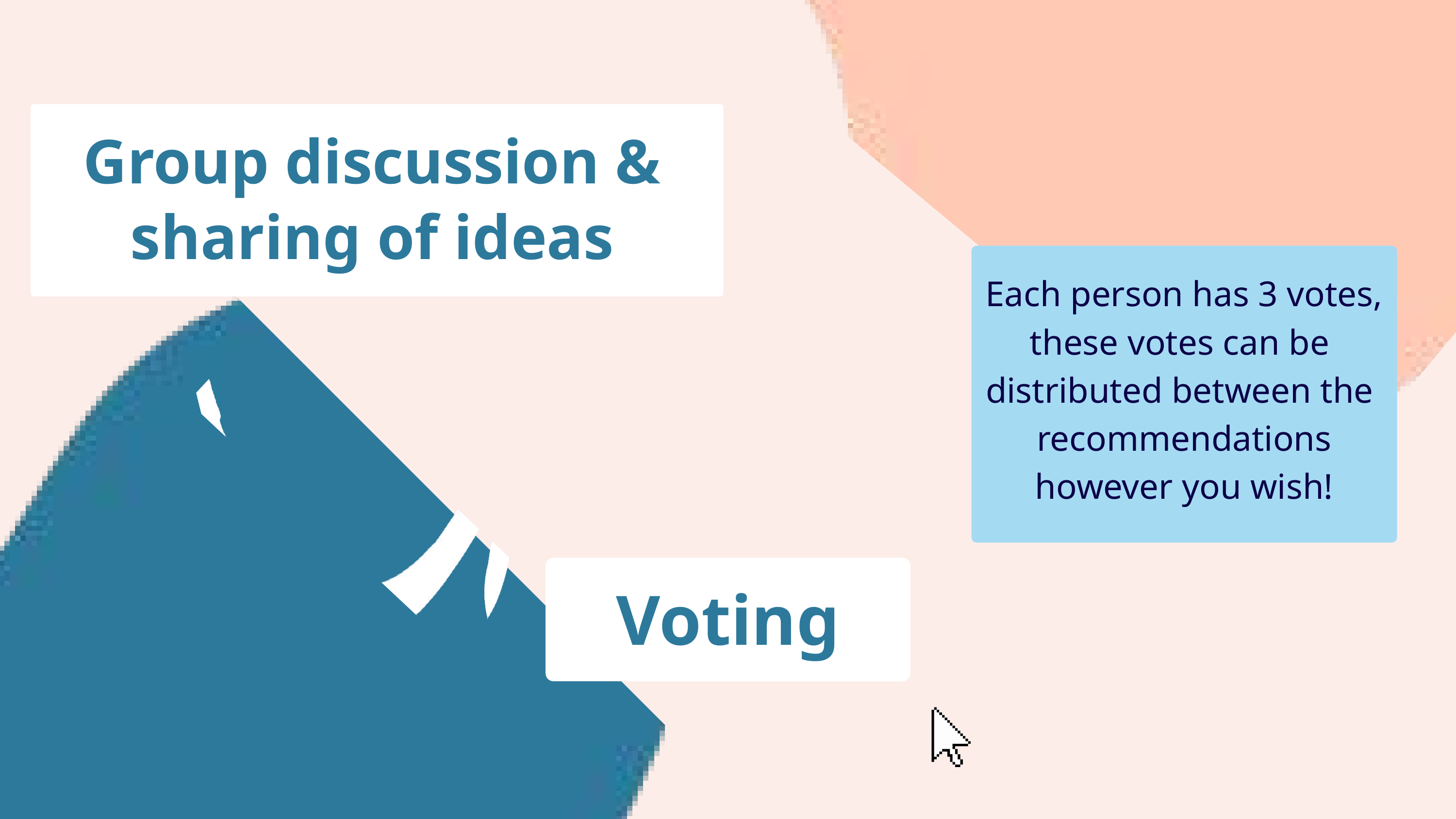

Group discussion & sharing of ideas
Each person has 3 votes, these votes can be
distributed between the
recommendations however you wish!
Voting

## Slide 18
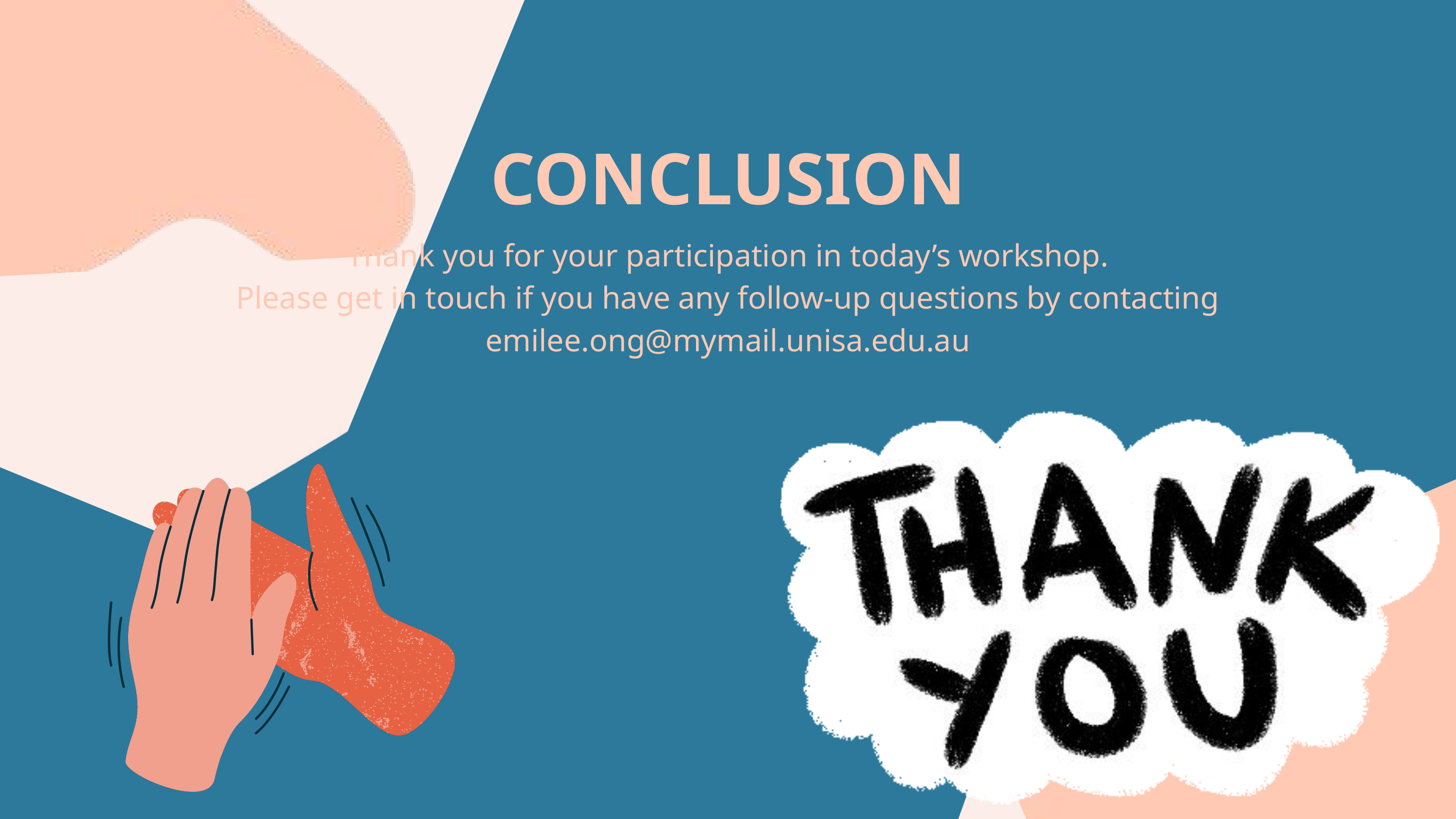

CONCLUSION
Thank you for your participation in today’s workshop.
Please get in touch if you have any follow-up questions by contacting emilee.ong@mymail.unisa.edu.au

## Slide 19
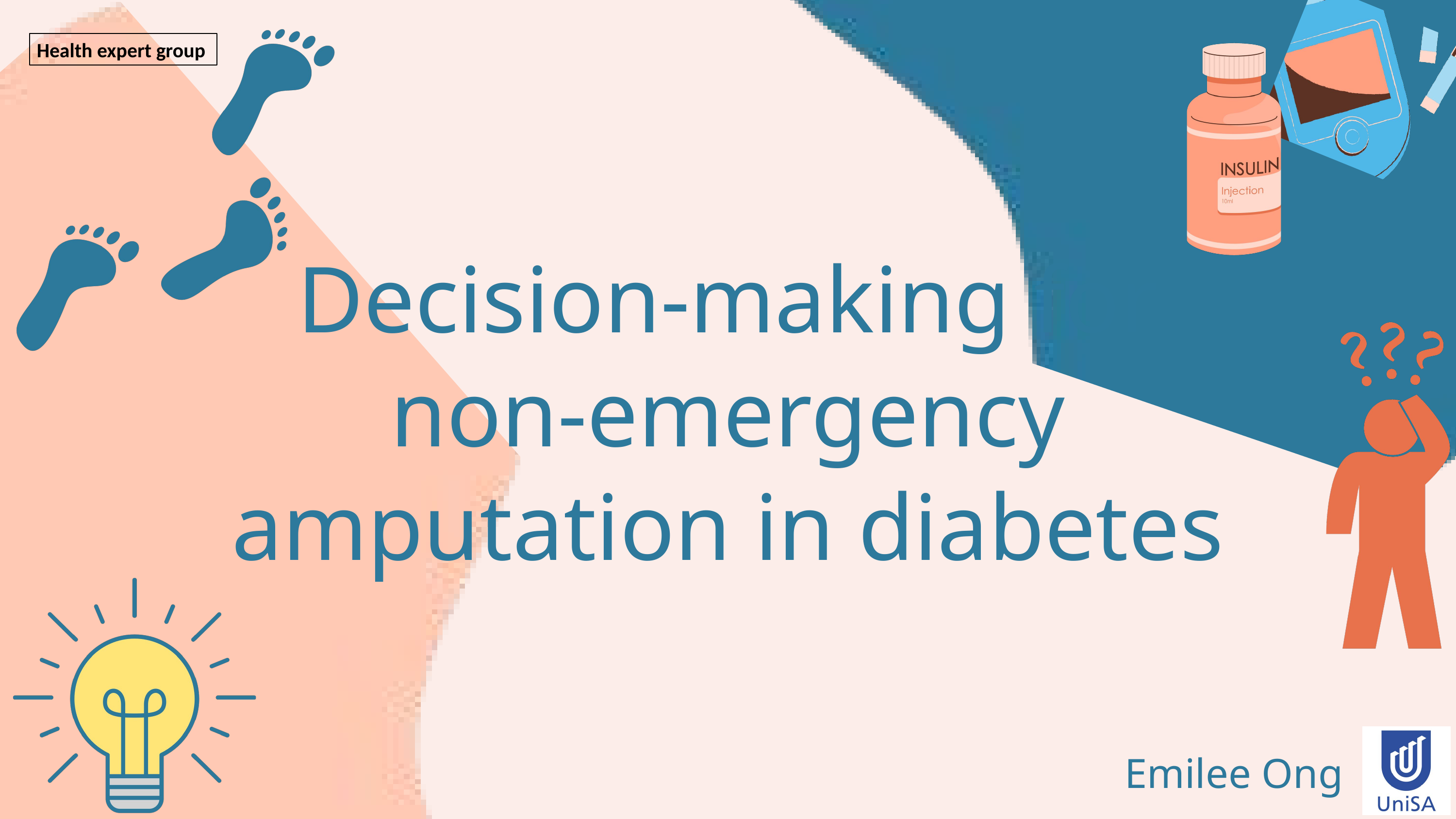

Health expert group
Decision-making for non-emergency amputation in diabetes
Emilee Ong

## Slide 20
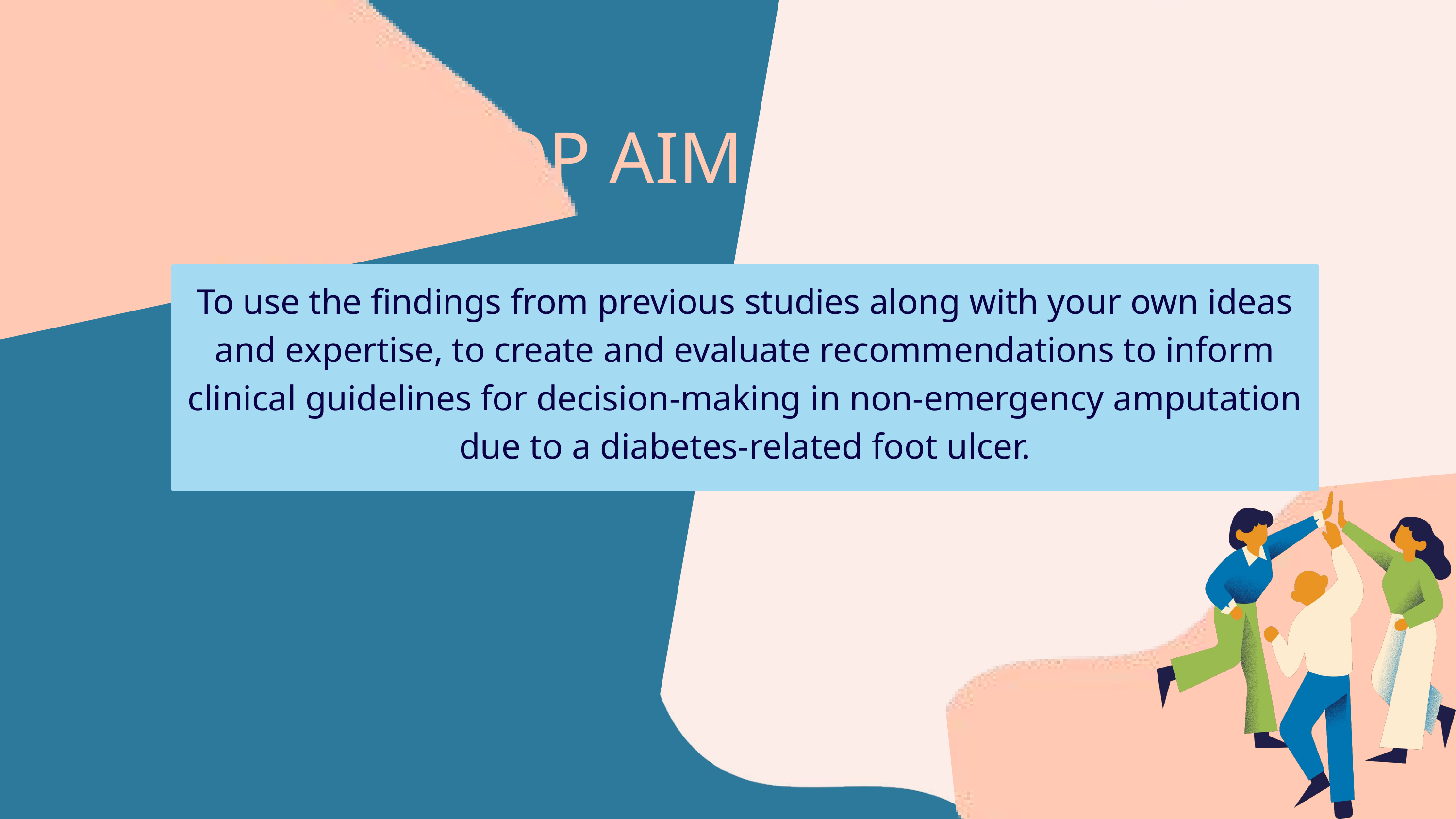

WORKSHOP AIM
To use the findings from previous studies along with your own ideas and expertise, to create and evaluate recommendations to inform clinical guidelines for decision-making in non-emergency amputation due to a diabetes-related foot ulcer.

## Slide 21
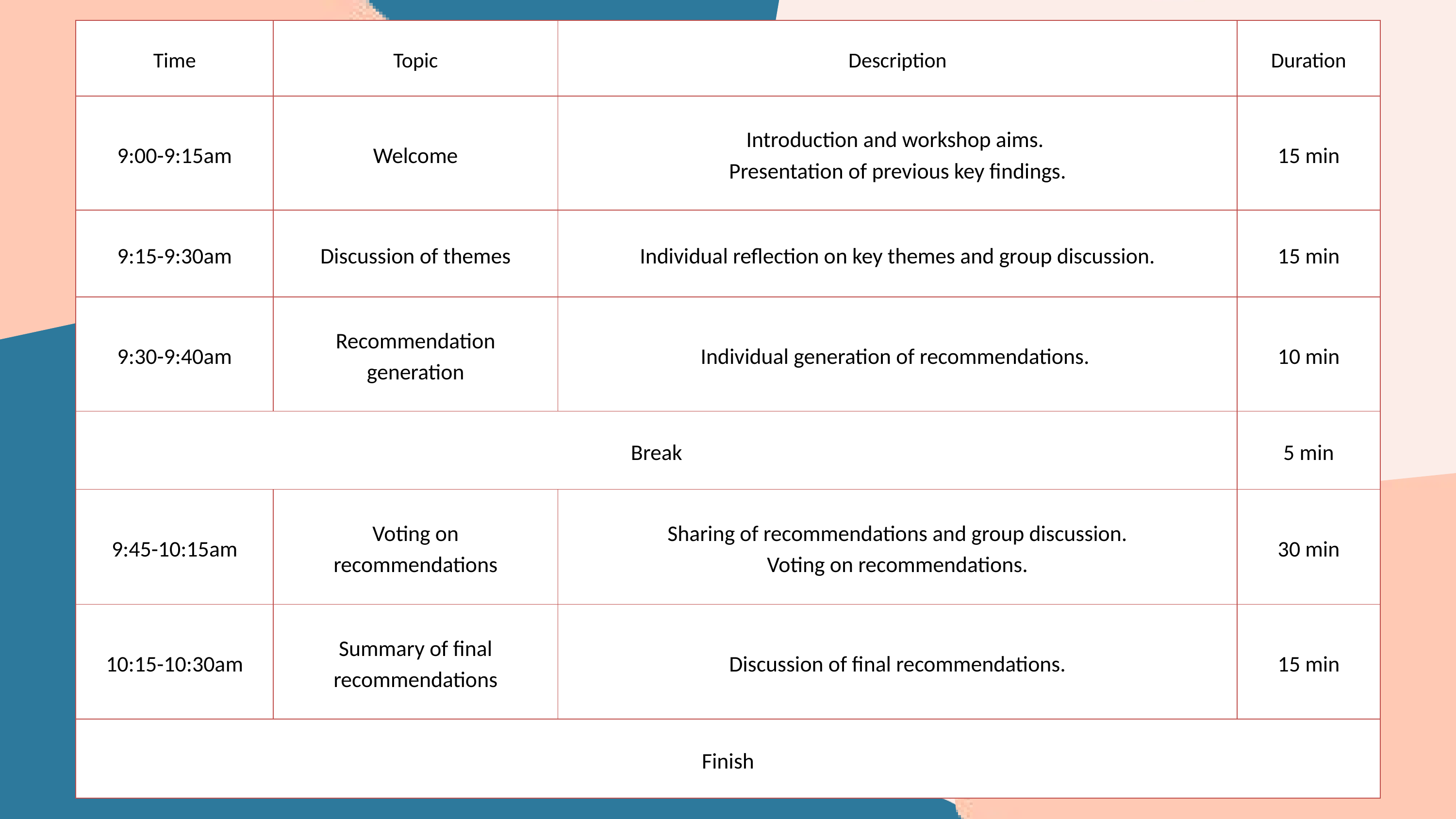

| Time | Topic | Description | Duration |
| --- | --- | --- | --- |
| 9:00-9:15am | Welcome | Introduction and workshop aims. Presentation of previous key findings. | 15 min |
| 9:15-9:30am | Discussion of themes | Individual reflection on key themes and group discussion. | 15 min |
| 9:30-9:40am | Recommendation generation | Individual generation of recommendations. | 10 min |
| Break | Break | Break | 5 min |
| 9:45-10:15am | Voting on recommendations | Sharing of recommendations and group discussion. Voting on recommendations. | 30 min |
| 10:15-10:30am | Summary of final recommendations | Discussion of final recommendations. | 15 min |
| Finish | Finish | Finish | Finish |

## Slide 22
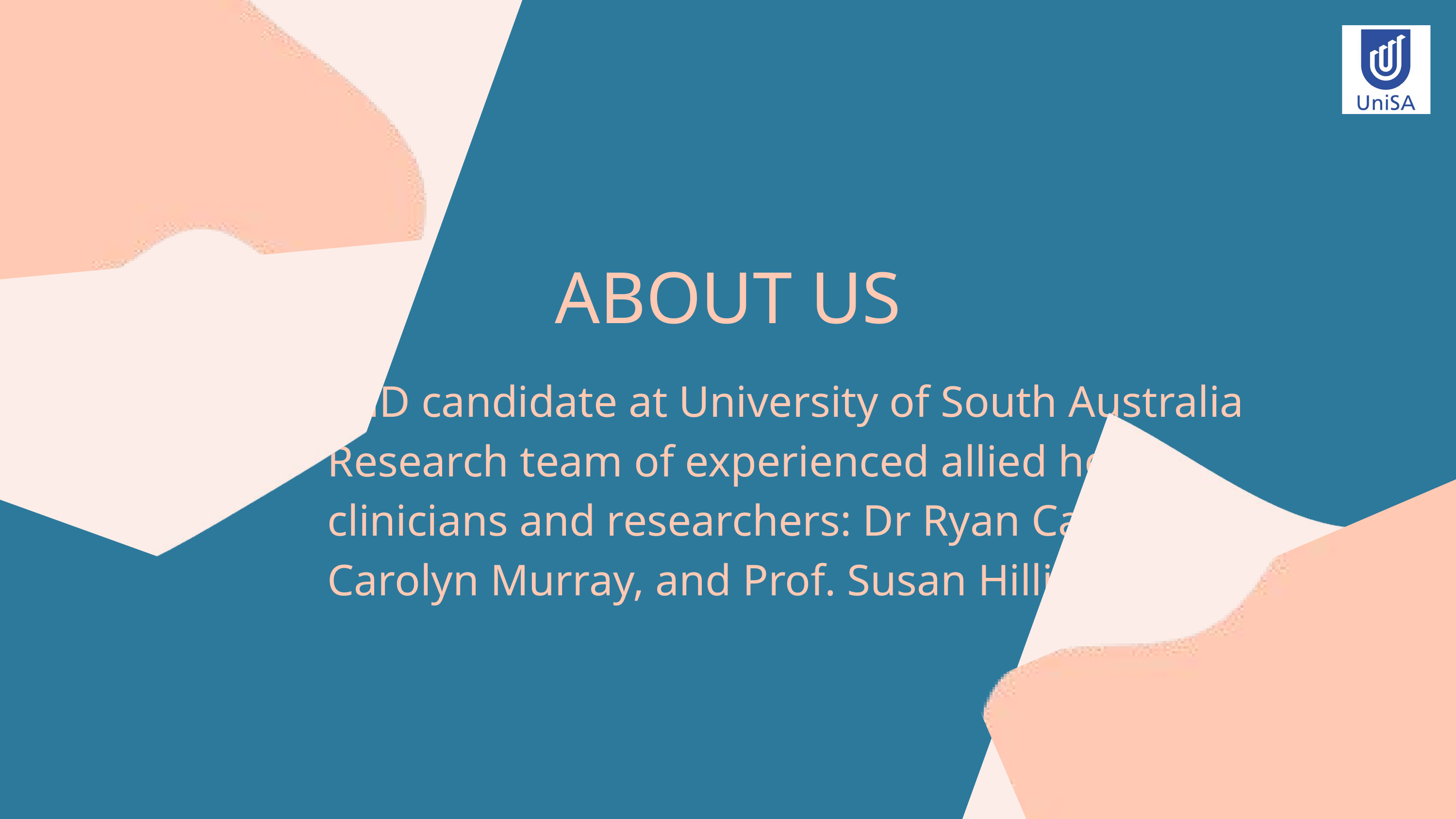

ABOUT US
PhD candidate at University of South Australia
Research team of experienced allied health clinicians and researchers: Dr Ryan Causby, Dr Carolyn Murray, and Prof. Susan Hillier

## Slide 23
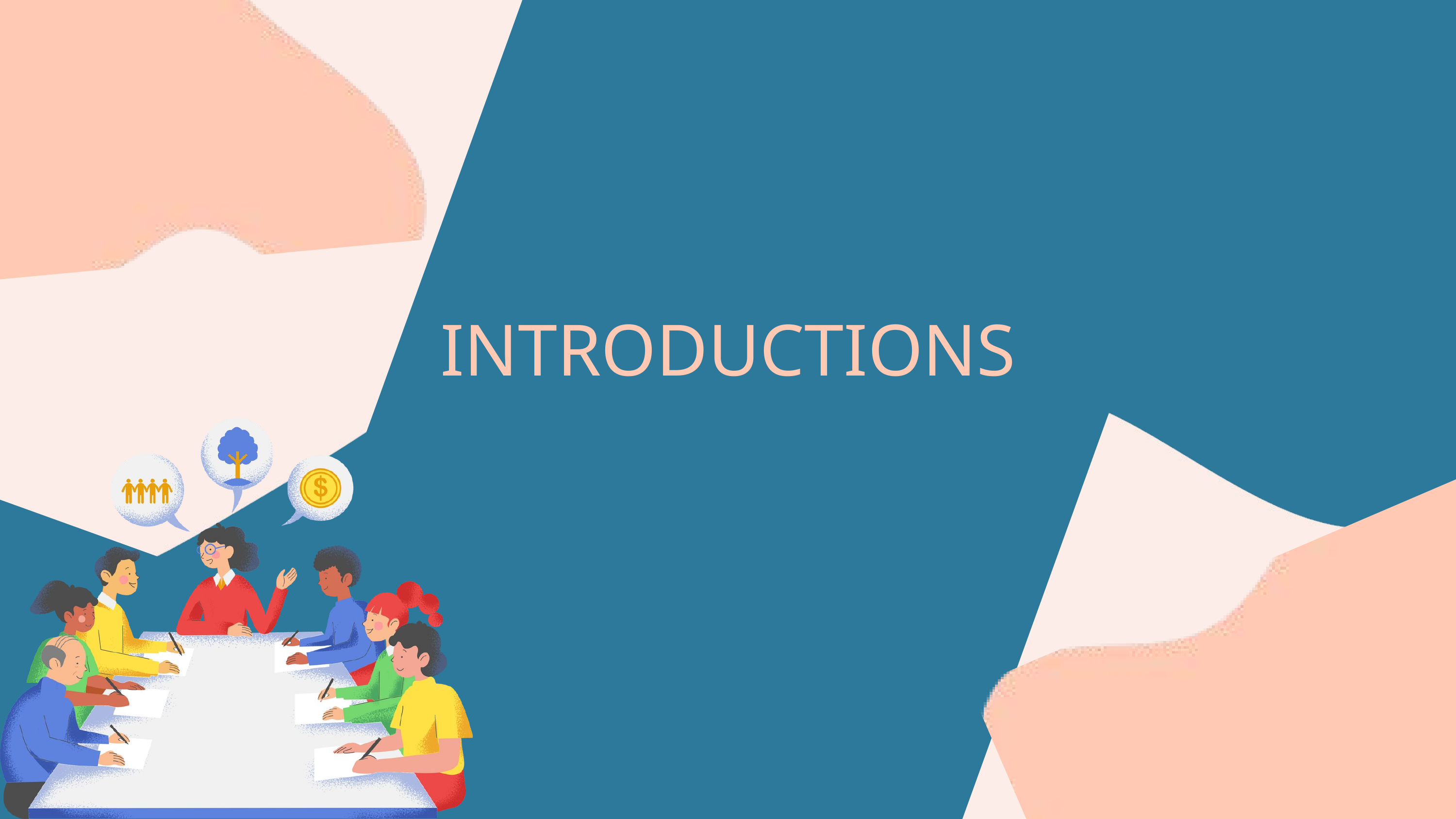

INTRODUCTIONS

## Slide 24
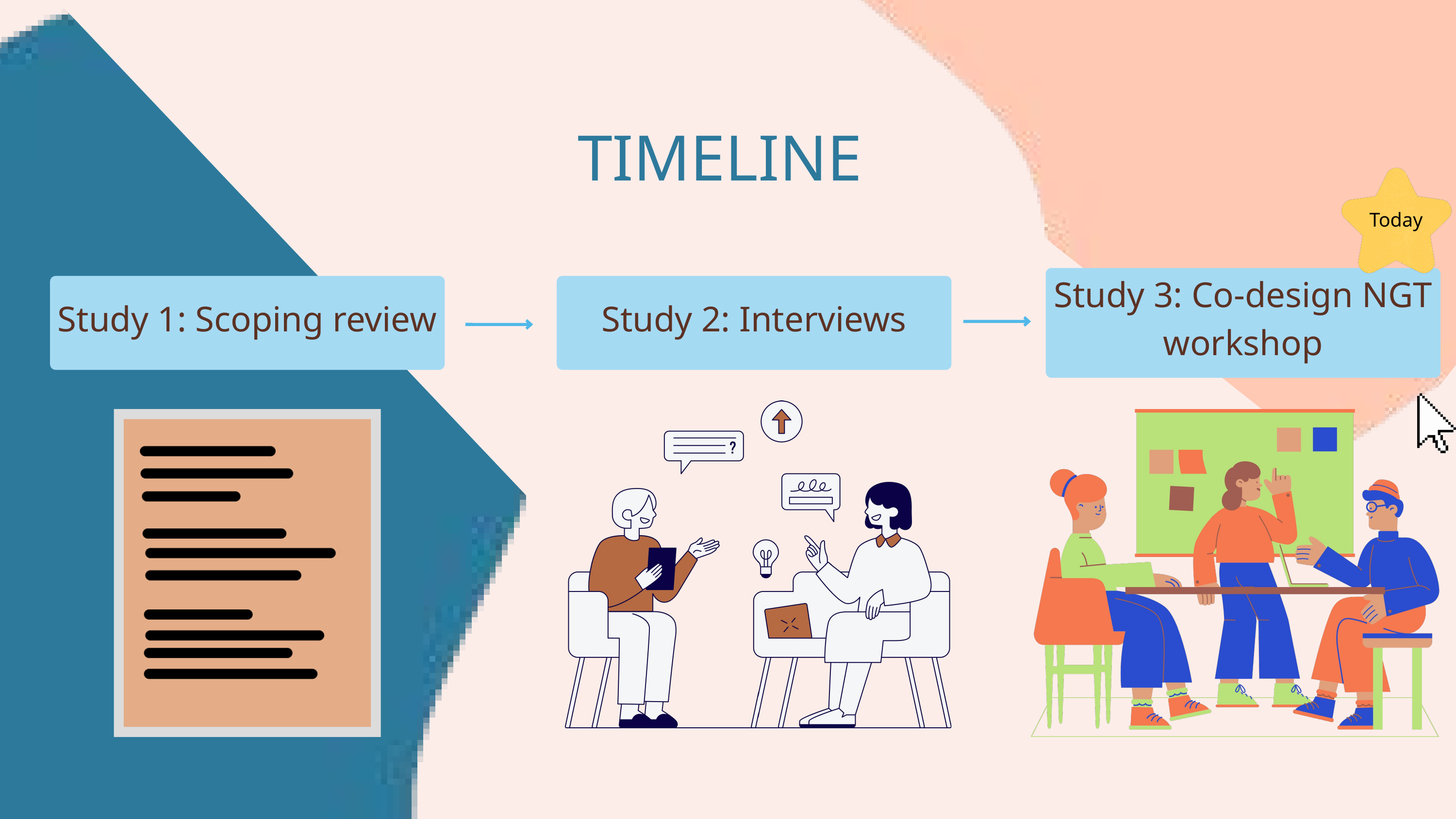

TIMELINE
Today
Study 3: Co-design NGT workshop
Study 1: Scoping review
Study 2: Interviews

## Slide 25
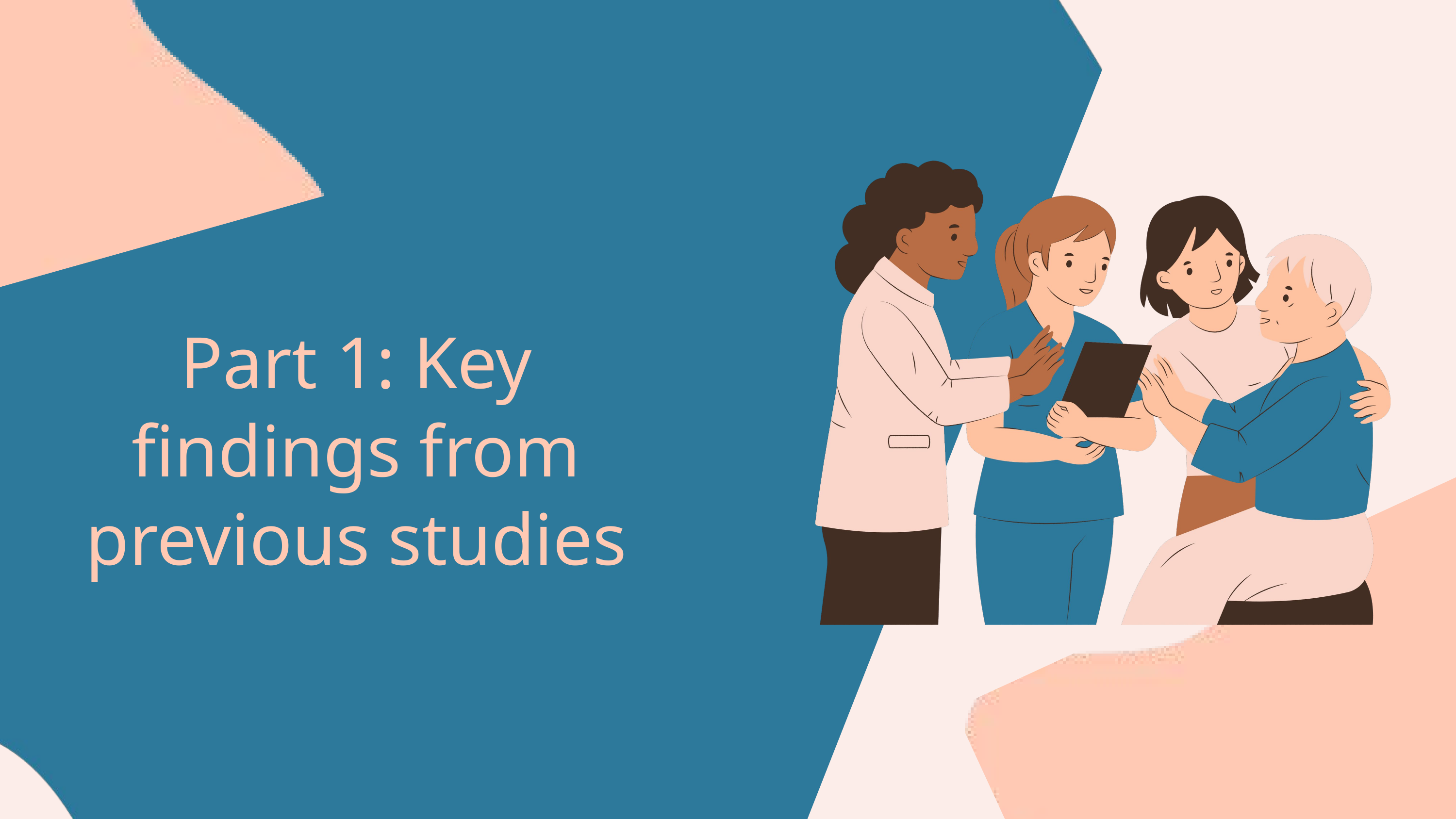

Part 1: Key findings from previous studies

## Slide 26
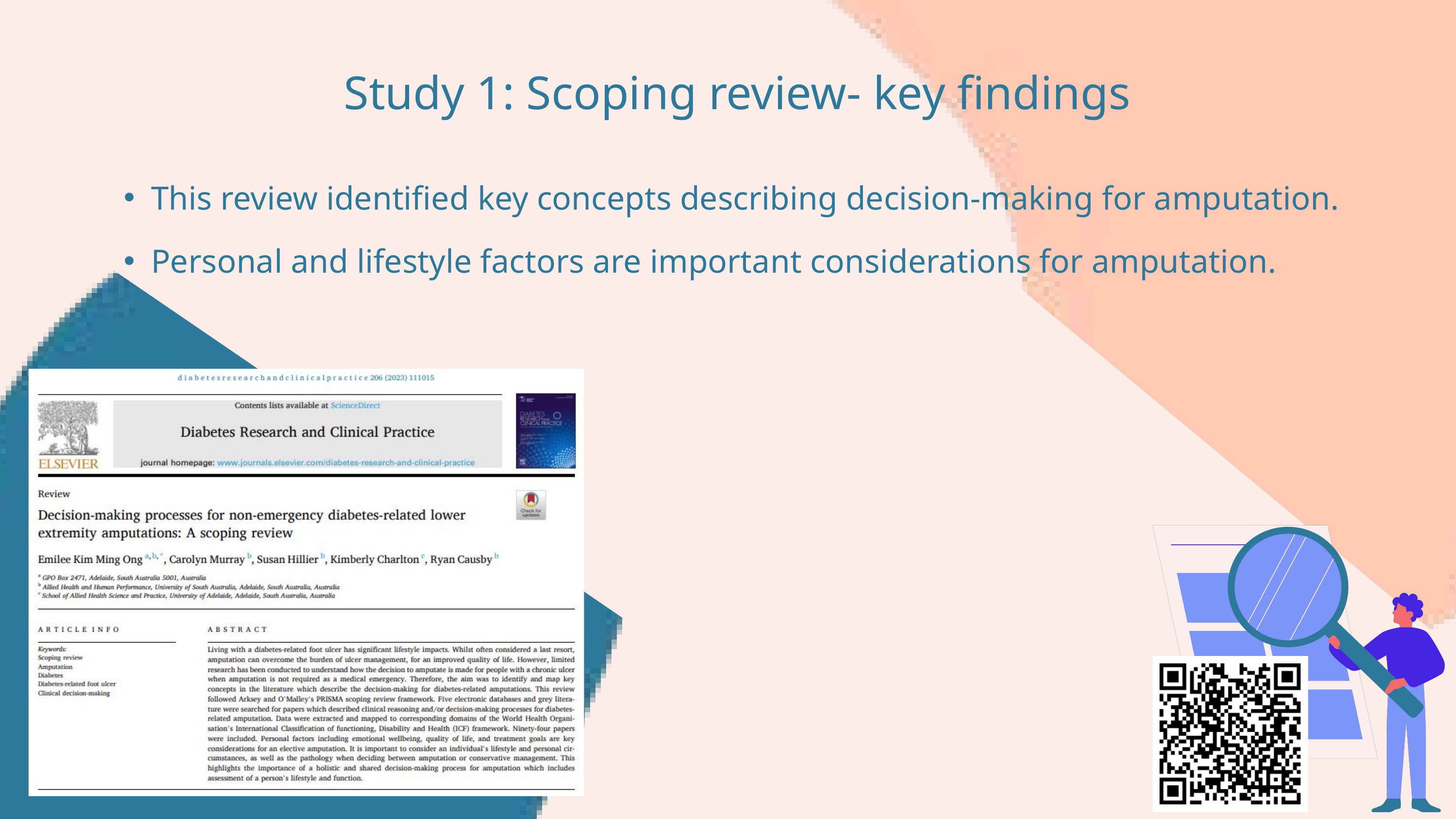

Study 1: Scoping review- key findings
This review identified key concepts describing decision-making for amputation.
Personal and lifestyle factors are important considerations for amputation.

## Slide 27
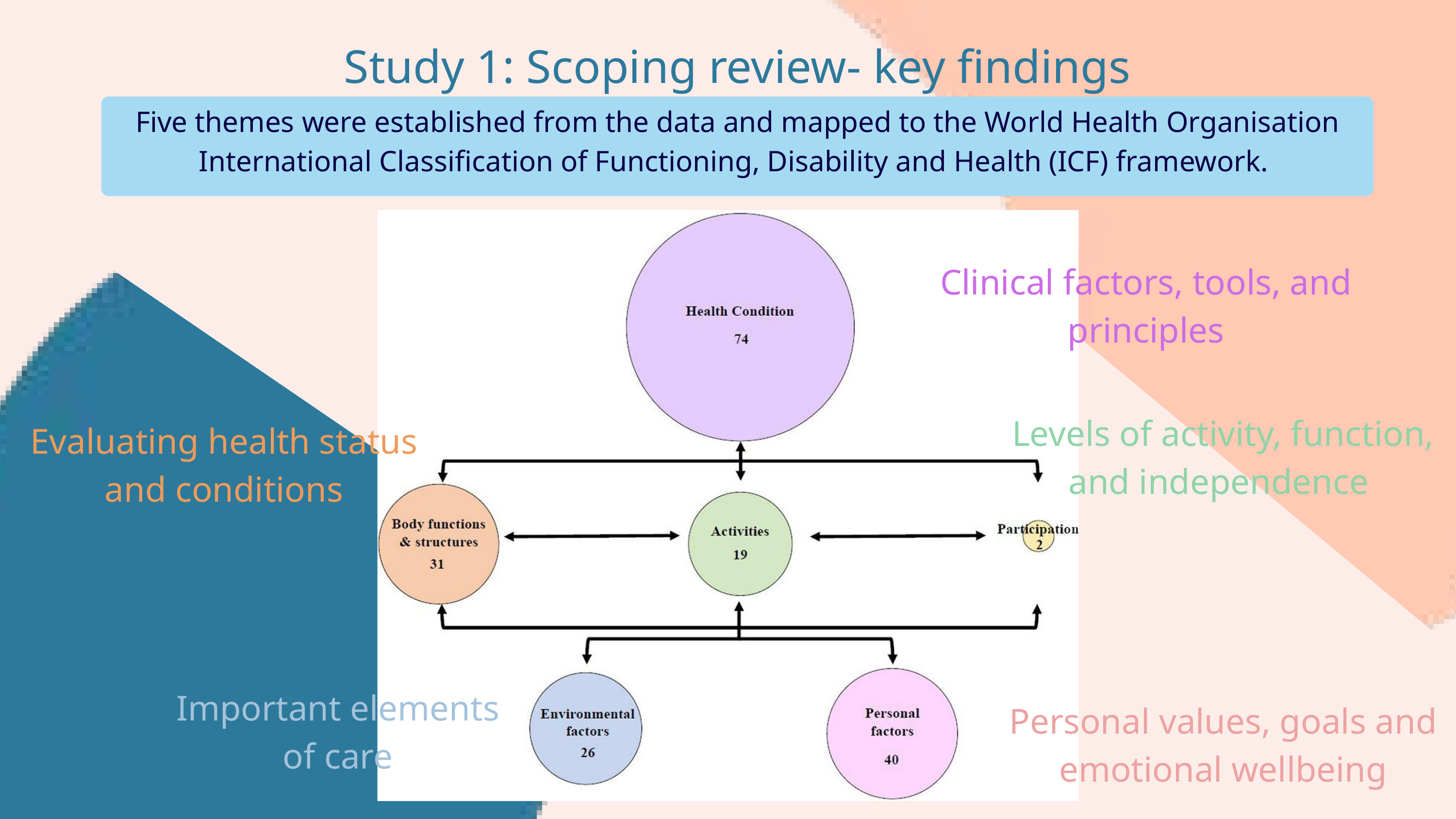

Study 1: Scoping review- key findings
Five themes were established from the data and mapped to the World Health Organisation International Classification of Functioning, Disability and Health (ICF) framework.
Clinical factors, tools, and principles
Levels of activity, function,
and independence
Evaluating health status
and conditions
Important elements of care
Personal values, goals and emotional wellbeing

## Slide 28
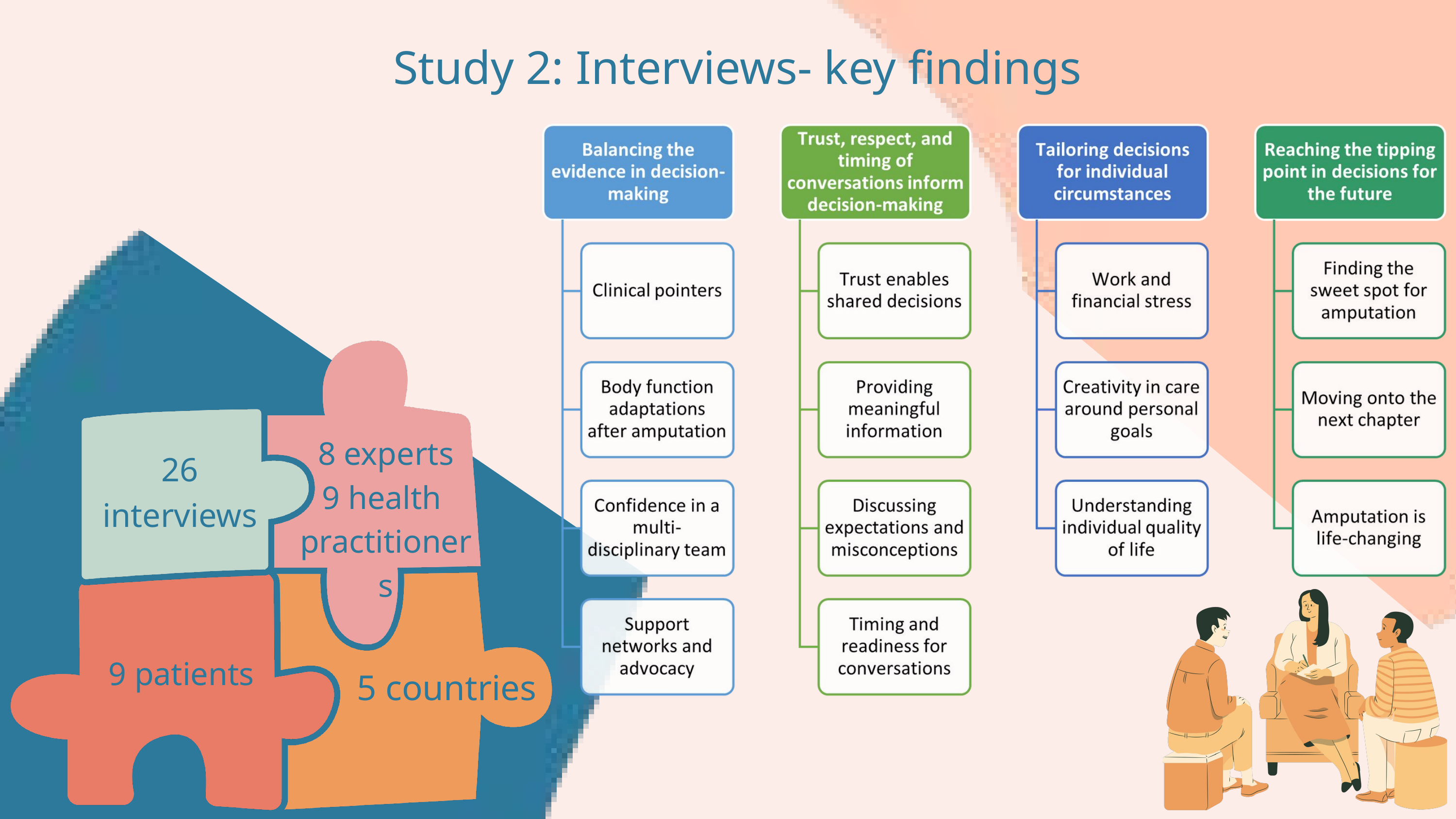

Study 2: Interviews- key findings
8 experts
9 health
practitioners
26 interviews
9 patients
5 countries

## Slide 29
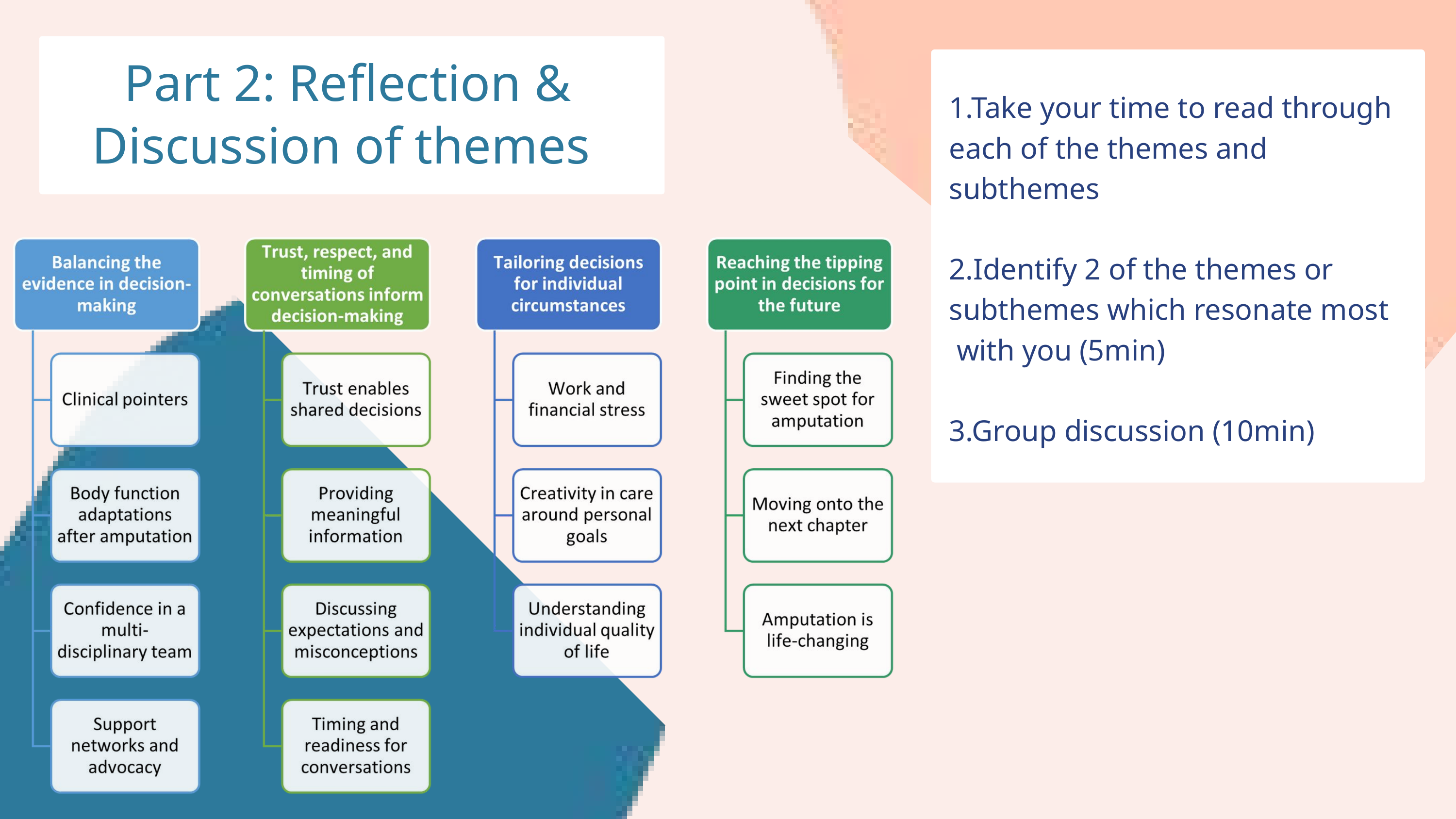

Part 2: Reflection & Discussion of themes
1.Take your time to read through
each of the themes and subthemes
2.Identify 2 of the themes or
subthemes which resonate most
 with you (5min)
3.Group discussion (10min)

## Slide 30
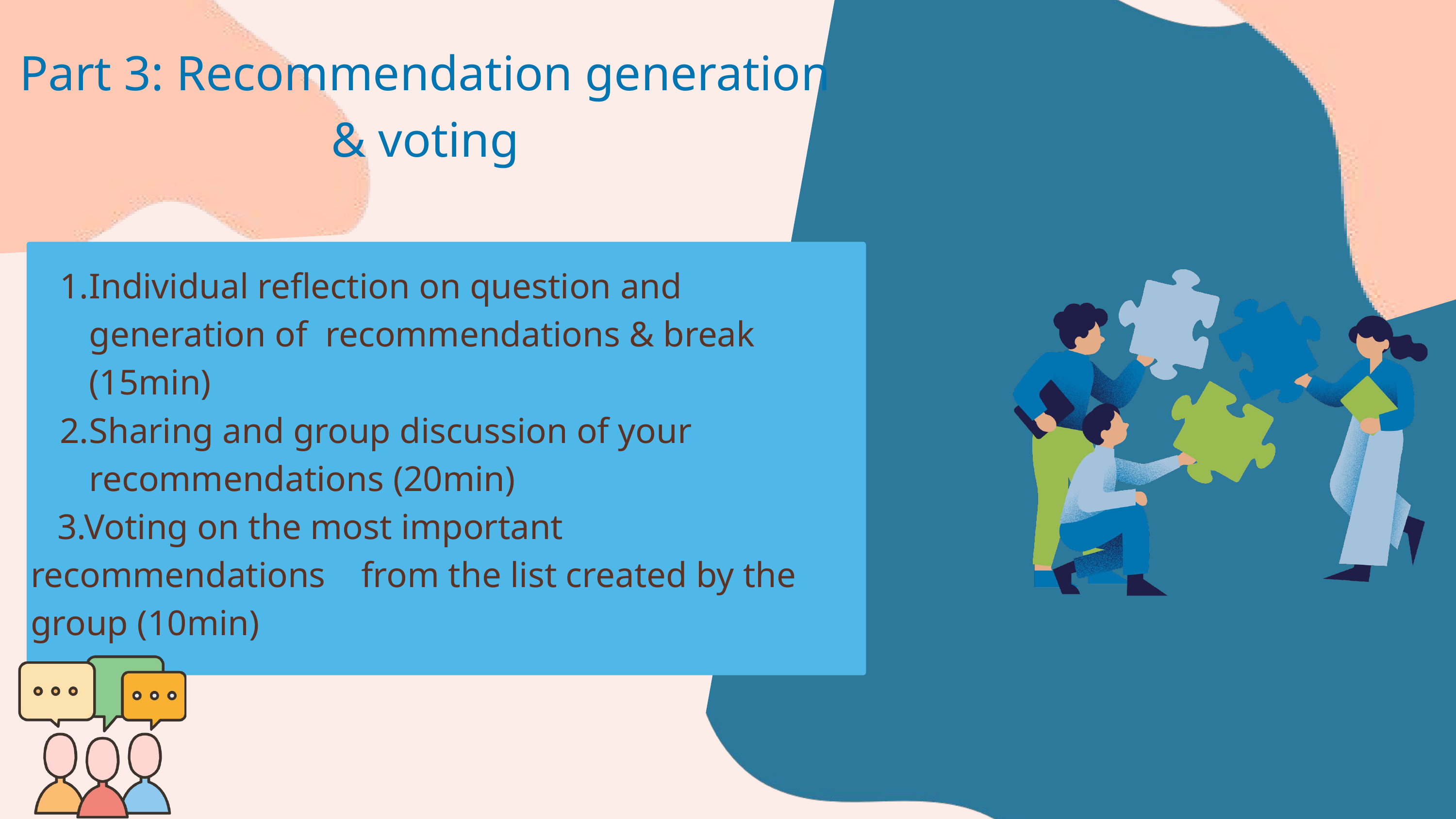

Part 3: Recommendation generation & voting
Individual reflection on question and generation of recommendations & break (15min)
Sharing and group discussion of your recommendations (20min)
 3.Voting on the most important recommendations from the list created by the group (10min)

## Slide 31
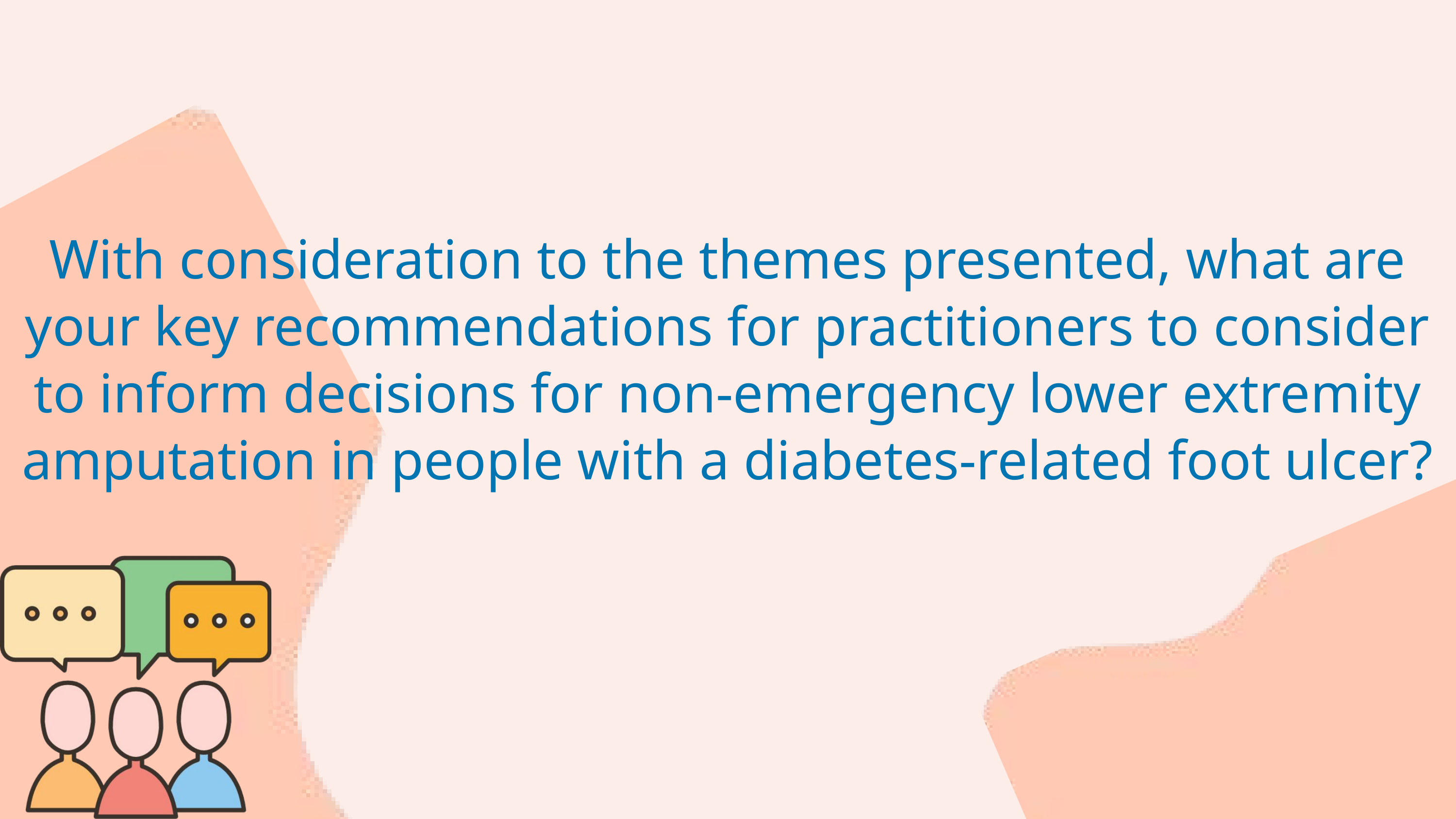

With consideration to the themes presented, what are your key recommendations for practitioners to consider to inform decisions for non-emergency lower extremity amputation in people with a diabetes-related foot ulcer?

## Slide 32
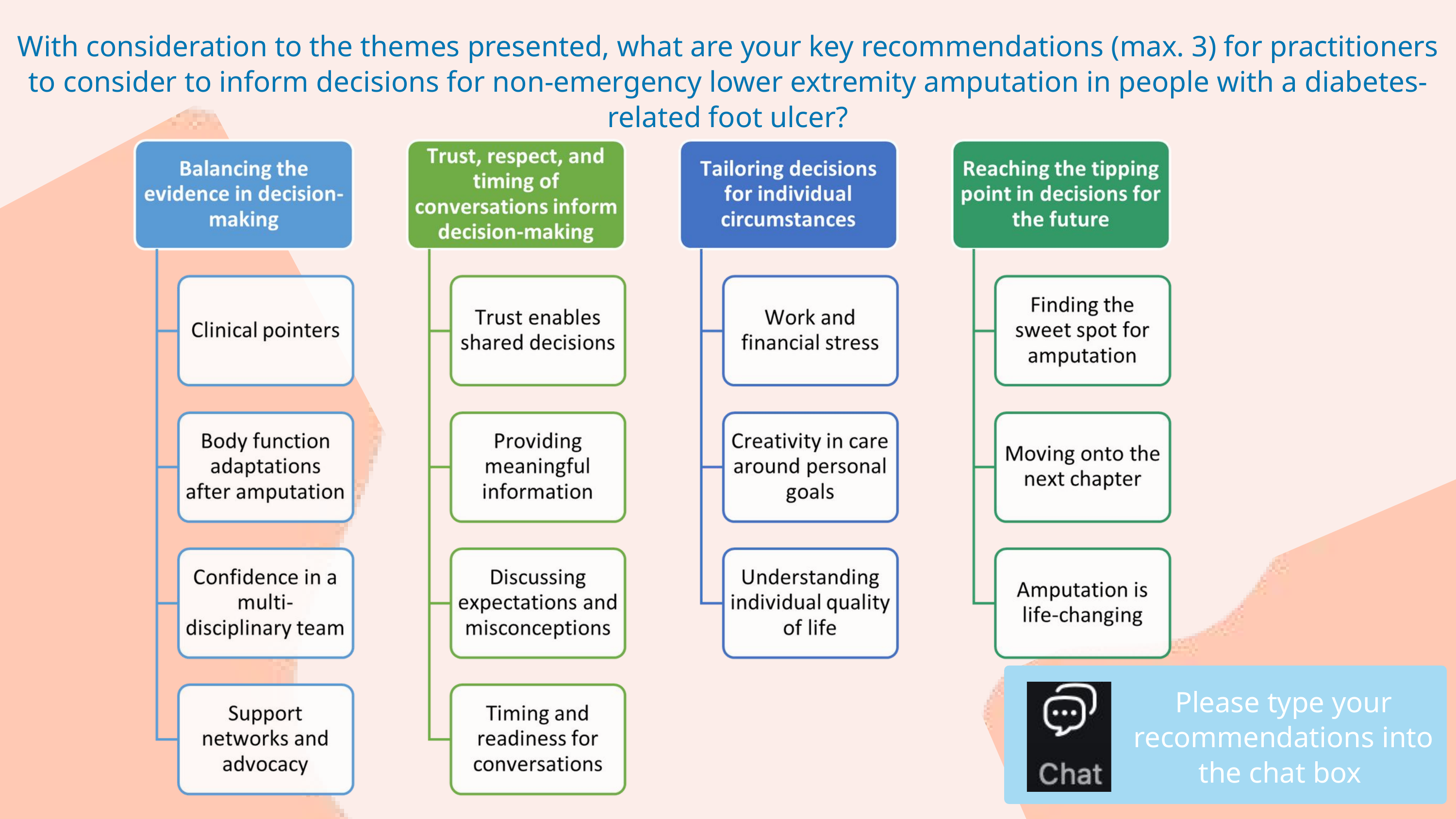

With consideration to the themes presented, what are your key recommendations (max. 3) for practitioners to consider to inform decisions for non-emergency lower extremity amputation in people with a diabetes-related foot ulcer?
Please type your recommendations into the chat box

## Slide 33
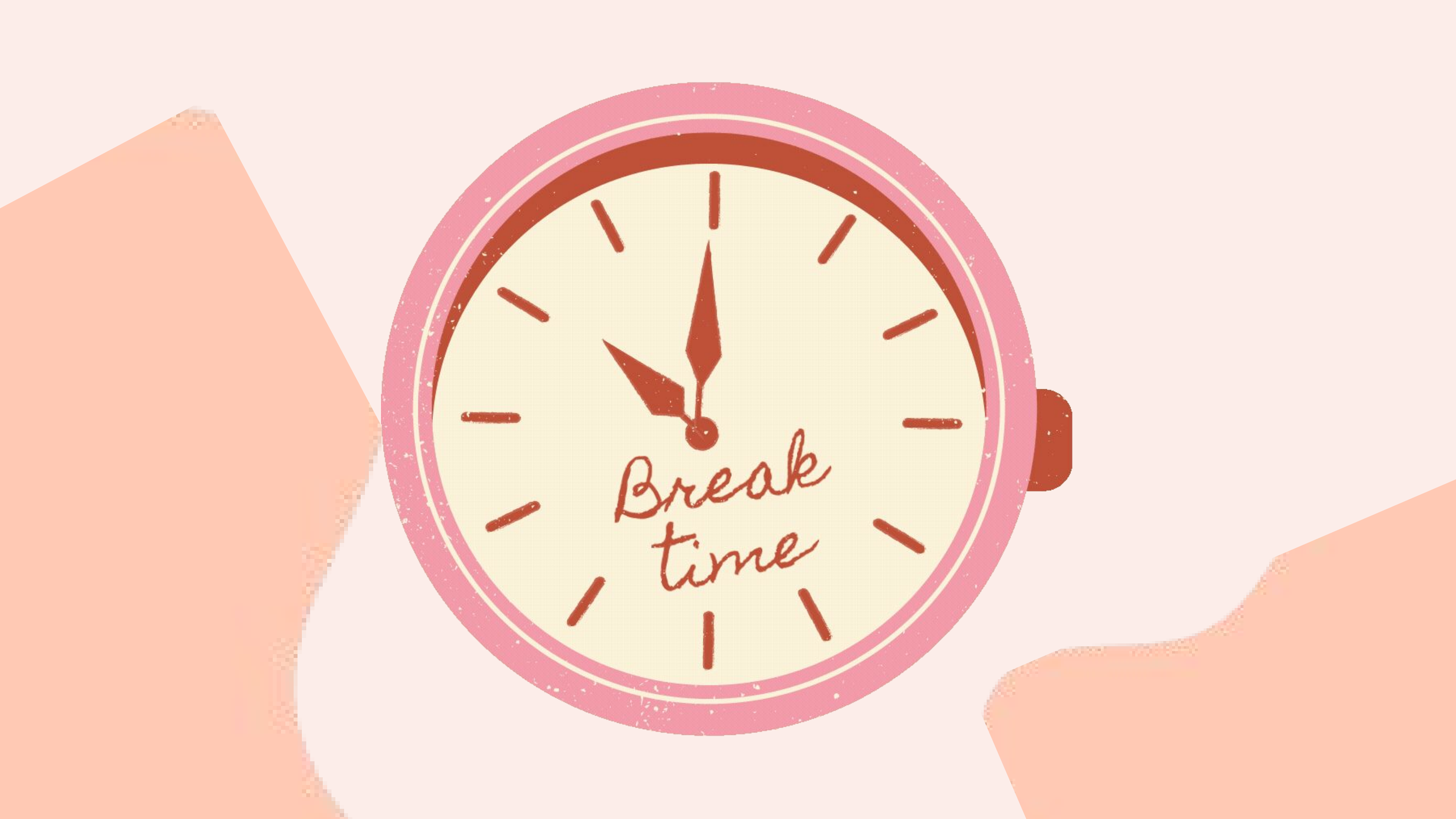

## Slide 34
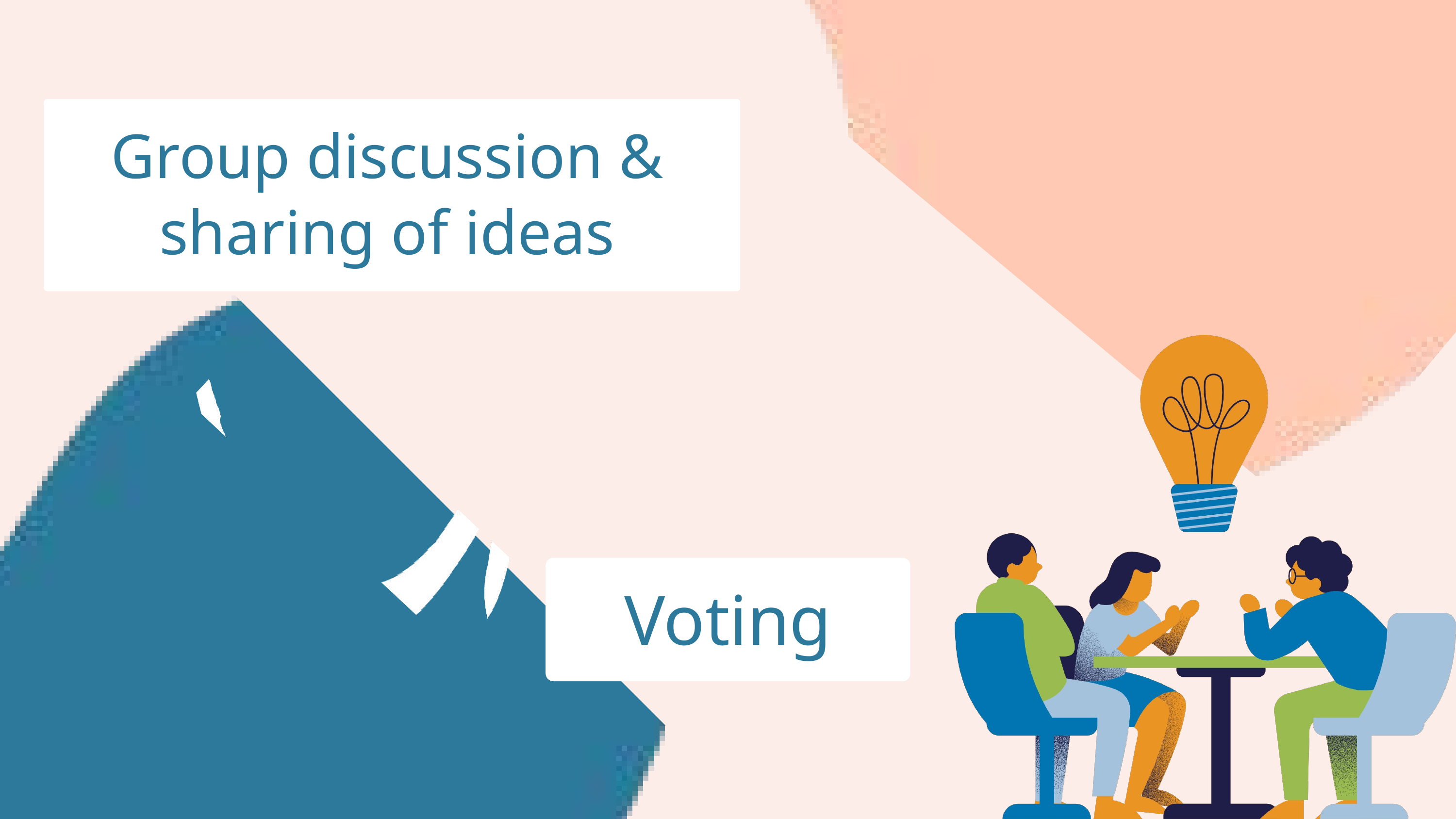

Group discussion & sharing of ideas
Voting

## Slide 35
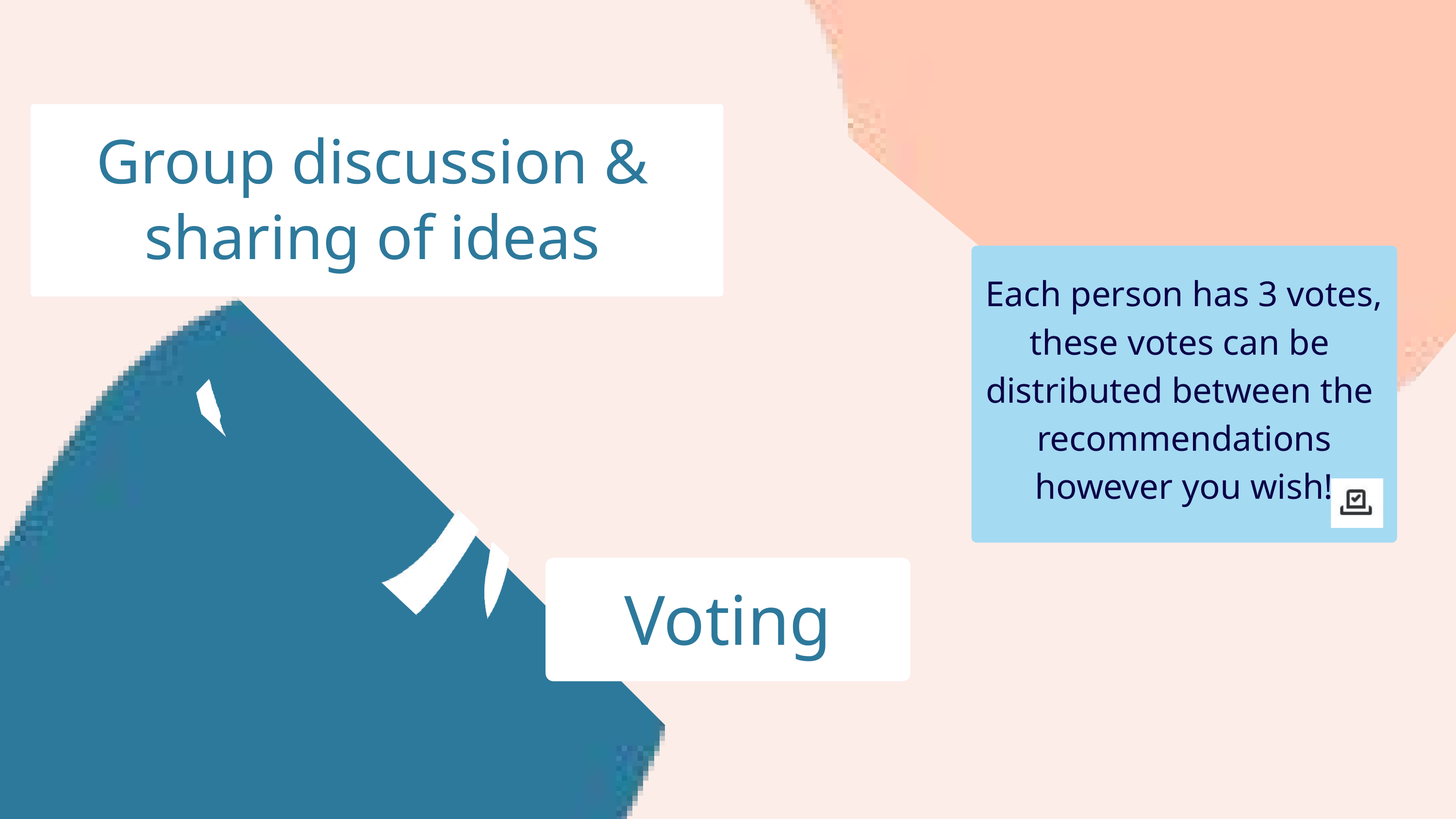

Group discussion & sharing of ideas
Each person has 3 votes, these votes can be
distributed between the
recommendations however you wish!
Voting

## Slide 36
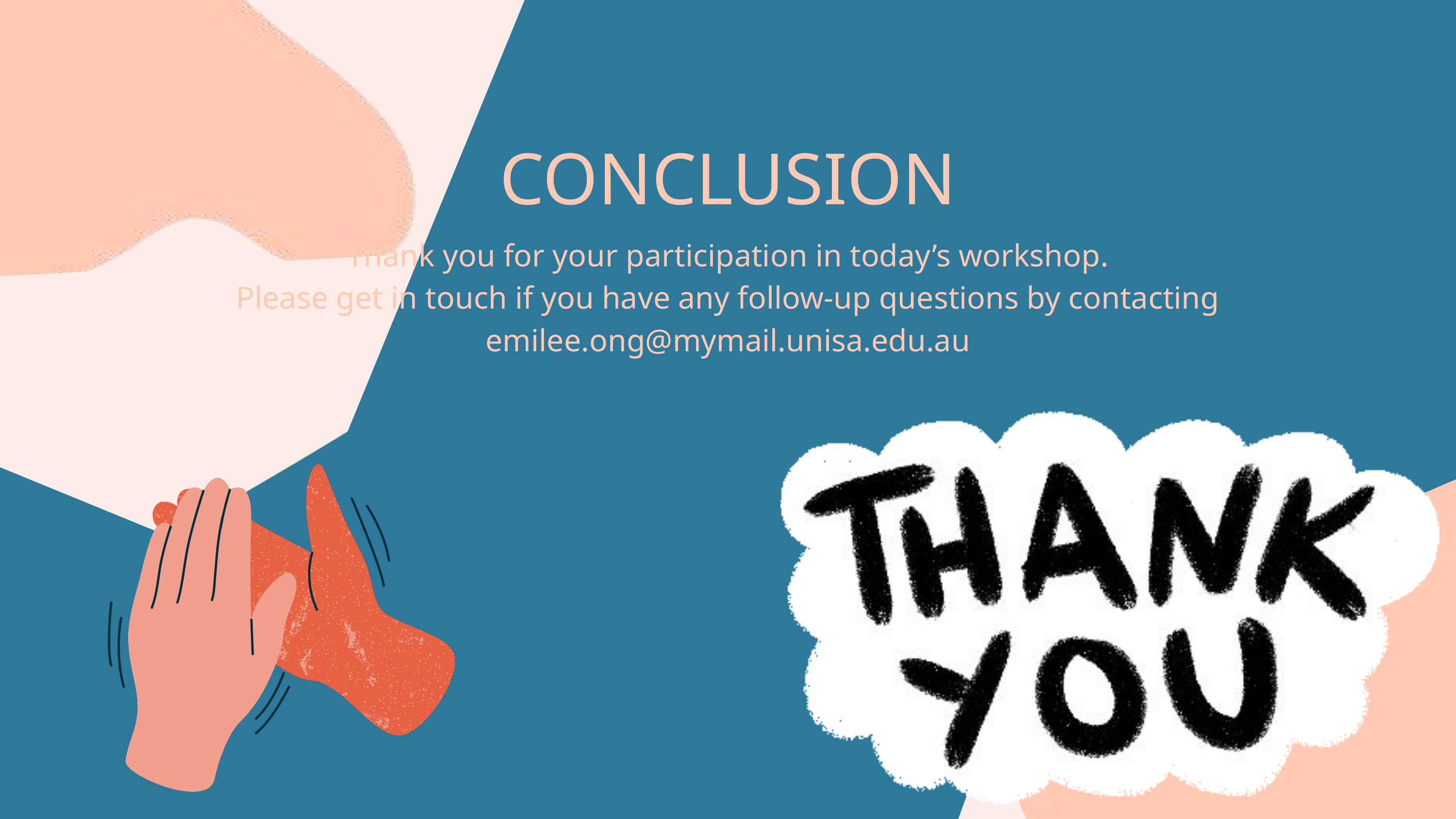

CONCLUSION
Thank you for your participation in today’s workshop.
Please get in touch if you have any follow-up questions by contacting emilee.ong@mymail.unisa.edu.au
